# Dynamic Behavior of the Glassy and Supercooled Liquid States of Aceclofenac Assessed by Dielectric and Calorimetric Techniques

**DOI:** 10.3390/molecules30030681

**Published:** 2025-02-04

**Authors:** M. Teresa Viciosa, Joaquim J. Moura Ramos, Ana Rosa Garcia, Hermínio P. Diogo

**Affiliations:** 1 Centro de Química Estrutural, Institute of Molecular Sciences, Instituto Superior Técnico, Universidade de Lisboa, Av. Rovisco Pais, 1049-001 Lisboa, Portugal; mouraramos@tecnico.ulisboa.pt (J.J.M.R.); argarcia@ualg.pt (A.R.G.); 2 Departamento de Química e Farmácia, Faculdade de Ciências e Tecnologia, Universidade do Algarve, Campus de Gambelas, 8000-139 Faro, Portugal

**Keywords:** aceclofenac, amorphous, glass transition, dipolar relaxations, conductivity

## Abstract

Aceclofenac (ACF), a non-steroidal anti-inflammatory drug, was obtained in its amorphous state by cooling from melt. The glass transition was investigated using dielectric and calorimetric techniques, namely, dielectric relaxation spectroscopy (DRS), thermally stimulated depolarization currents (TSDC), and conventional and temperature-modulated differential scanning calorimetry (DSC and TM-DSC). The dynamic behavior in both the glassy and supercooled liquid states revealed multiple relaxation processes. Well below the glass transition, DRS was able to resolve two secondary relaxations, γ and β, the latter of which was also detectable by TSDC. The kinetic parameters indicated that both processes are associated with localized motions within the molecule. The main (α) relaxation was clearly observed by DRS and TSDC, and results from both techniques confirmed a non-Arrhenian temperature dependence of the relaxation times. However, the glass transition temperature (T_g_) extrapolated from DRS data significantly differed from that obtained via TSDC, which in turn showed reasonable agreement with the calorimetric T_g_ (T_g-DSC_ = 9.2 °C). The values of the fragility index calculated by the three experimental techniques converged in attributing the character of a moderately fragile glass former to ACF. Above the α relaxation, TSDC showed a well-defined peak. In DRS, after “removing” the high-conductivity contribution using ε’ derivative analysis, a peak with shape parameters *α*_HN_ = *β*_HN_ = 1 was also detected. The origin of these peaks, found in the full supercooled liquid state, has been discussed in the context of structural and dynamic heterogeneity. This is supported by significant differences observed between the FTIR spectra of the amorphous and crystalline samples, which are likely related to aggregation differences resulting from variations in the hydrogen bonds between the two phases. Additionally, the pronounced decoupling between translational and relaxational motions, as deduced from the low value of the fractional exponent *x* = 0.72, derived from the fractional Debye–Stokes–Einstein (FDSE) relationship, further supports this interpretation.

## 1. Introduction

Aceclofenac (ACF), chemical name 2-[(2,6-dichlorophenyl)amine] phenylacetoxyacetic acid, is a nonsteroidal anti-inflammatory drug (NSAID) commonly used to treat rheumatoid arthritis, osteoarthritis, and various inflammatory disorders, including odontalgia [1,2,3].

ACF was approved by the European Medicines Agency in the crystalline form I in 1990 [4]. It belongs to class II of the biopharmaceutics classification system, mainly due to its poor water solubility of 0.0035 mg∙mL^−1^ [5] and 58.67 μg∙mL^−1^ [6]. To improve solubility and the dissolution rate, a wide variety of formulations have been developed to date. Given its good absorption by the gastrointestinal tract, the oral route has been the most widely used method for administering ACF for several decades. Consequently, many formulations have been developed based on this, such as cocrystals [7], inclusion complexes [5], and salts [8]. However, due to various factors, including low solubility (which leads to inadequate absorption), chemical instability (since ACF can degrade into diclofenac), and the side effects associated with long-term administration, the topical route is currently being explored as a relevant alternative or complementary approach. In this context, many of the formulations under investigation involve the design of nanometer-scale transport systems, which offer advantages in terms of their ability to cross transdermal barriers and provide faster and more localized action. Examples of these approaches include oil-in-water nano-emulsions [9] and nano-suspensions [10].

In many of these formulations, the physical state of ACF can be different from the crystalline state. This is, for instance, the case of the amorphous form obtained in polymeric [11,12,13] and silica nanoparticles [14] or in solid dispersions [15] just to refer a few examples.

The lack of 3D order at long distances, characteristic of the amorphous state, results in an excess of Gibbs energy, which may contribute to higher solubility than that of the corresponding ordered crystalline structure [16,17]. The underlying instability is due to the fact that it is a non-equilibrium thermodynamic state, which can be modified through interaction with the surrounding matrix and/or by the confinement effect in nanometric dimensions.

To accelerate the optimization process of any of these amorphous ACF formulations, including, for instance, binary mixtures [18], solid formulations [19,20], or nanofibers [21], it seems essential to have thorough knowledge of the physicochemical characteristics of this state.

In this context, the main aim of this work was to assess the complex behavior of the amorphous form of ACF by evaluating its molecular mobility over a wide range of frequencies (from ~10^−3^ to 10^6^ Hz) and temperatures, covering both the glassy and the supercooled liquid states. To this end, dielectric relaxation spectroscopy (DRS) and thermally stimulated depolarization (TSDC) techniques were extensively used. The obtained information has been combined to gain a deeper understanding of the features of the amorphous bulk material, along with data provided by calorimetric and FTIR analyses, conventional techniques that are part of the portfolio used to characterize pharmaceuticals.

In future research, DRS and TSDC can be used as thermoanalytical techniques to investigate more complex systems, such as binary mixtures. By analyzing changes in the dynamic behavior of the bulk active pharmaceutical ingredient (API) compared to the behavior in a mixture, it may be possible to establish a correlation with the corresponding dissolution kinetics. Based on this knowledge, the manipulation of molecular mobility could allow optimized dissolution while also forecasting the physical stability and shelf life of drugs through dynamic insights provided by these techniques.

## 2. Results

### 2.1. Differential Scanning Calorimetry

Upon heating at 5 °C∙min^−1^ from ~−20 up to 170 °C (see Figure S1 in the Supplementary Information), the melting peak was observed at 152.6 °C, with melting enthalpy Δ*H_m_* = 128.2 J∙g^−1^, which is in good agreement with data reported in literature for polymorph I [22] (see Figure S1 in the Supplementary Information). On cooling and subsequent heating at 10 °C∙min^−1^, only the glass transition signal was observed, evidencing the full amorphization of this compound and the good glass-forming ability. The glass transition temperature (onset) determined on heating was 12.6 °C; it should be stressed that the value of the glass transition temperature is sensitive to small modifications of the amorphization procedure.

The experiments were programmed in such a way that the cooling rate of a given experiment was equal in absolute value to the rate of heating in the experiment that followed it, that is, |*q−/q+*| = 1.

A not yet described polymorph of ACF, obtained by cold crystallization and with a melting temperature lower than that of the original, was detected under conditions that are not completely reproducible; more details are given in the Supplementary Information (SI).

**Figure 1:**
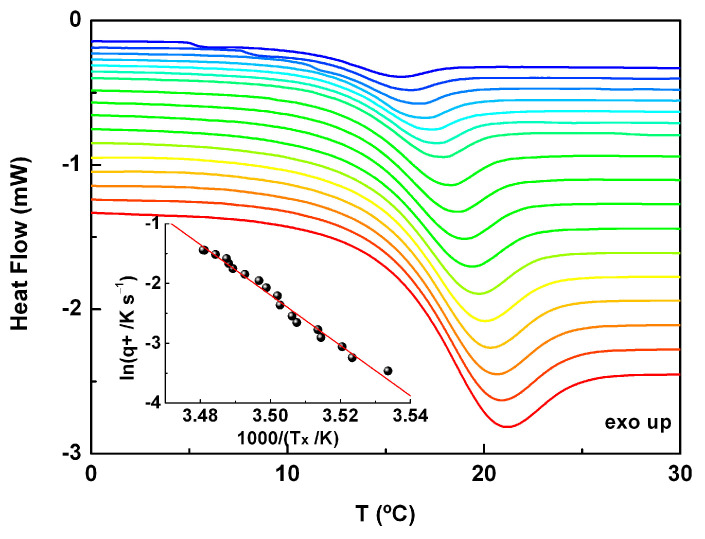
Thermograms obtained for amorphous ACF at heating rates (*q^+^*) varying from 2 (blue line) to 14 (red line) °C∙min^−1^ (see more details in Table S1 of the Supplementary Information). Inset: lo garithm of the heating rate, *q*^+^, plotted as a function 1000/*T_x_*, where *T_x_* is the onset temperature of the glass transition signal obtained from heating (cooling/heating cycles were programmed keeping the same rate, i.e., |*q^−^/q^+^*| = 1).

The activation energy characterizing the dynamic glass transition can be determined from the cooling/heating rate temperature dependence. Considering the thermograms collected on heating from the glassy to the supercooled liquid state, the shift to higher temperatures of the glass transition can be related to the activation energy as:

(1) $$\frac{d\left(lnq^{+}\right)}{d\left(1/T_{x}\right)}=-\frac{E_{a}\left(T_{g}\right)}{R}\;,$$

where *q+* is the heating rate and *T_x_* (onset) the glass transition temperature. Then, a set of cooling/heating cycles was carried out at different rates such that for each cycle, we had |*q*−/*q*+| = 1. In these conditions, the Tool–Narayanaswamy–Moynihan phenomenological model could be applied [23,24,25]. Some representative heating thermograms obtained for heating rates varying from 2 to 14 °C∙min^−1^ are displayed in Figure 1; the inset shows *ln|q|* as a function of the values obtained for the onset of the glass transition temperature peak, *T_x_*, for all the heating rates. The activation energy estimated from these results was 349.4 kJ∙mol^−1^ (correlation coefficient R^2^ = 0.986), and the average of the Δ*C_p_* values was 0.46 ± 0.02 J∙(g·°C)^−1^.

The fragility index [26,27], which indicates a measure of the degree of deviation from the Arrhenius-type temperature dependence at *T_g_*, can be estimated as:

(2) $$m=\frac{E_{a}\left(T_{g}\right)}{2.303RT_{g}}\;,$$

which leads to *m* = 64, allowing aceclofenac to be classified as a moderately fragile glass former comparable to indapamide (*m* = 66) [28].

**Figure 2:**
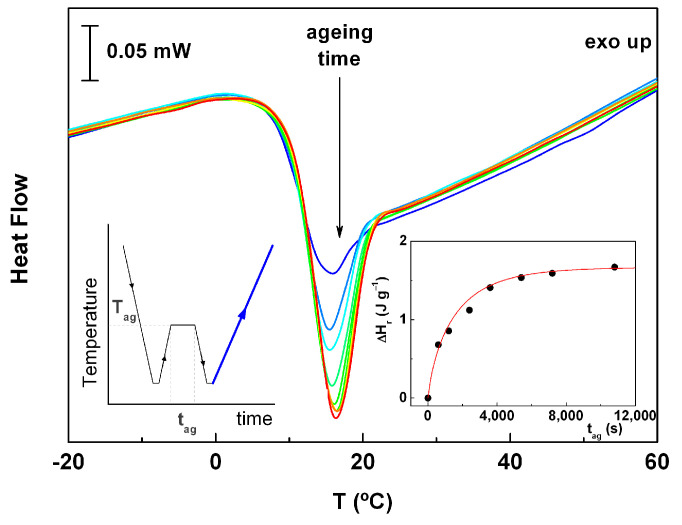
Thermograms obtained on heating at 5 °C∙min^−1^ under modulated conditions (amplitude = 0.66 °C, period = 50 s) for unaged sample and aged at different times: 10, 20, 40, 60, 90, 120, 180 and 210 min; the left-hand-side inset is a schematic representation of the experimental procedure used to induce physical aging of the sample. The right-hand-side inset displays the Δ*H_r_* as a function of the aging time; the line represents the best-fitted line obtained using Equation (3).

To evaluate the sensitivity of ACF to physical aging, a set of experiments involving different aging times was performed at T_ag_ = 5 °C (see the inset on the left-hand-side of Figure 2 schematically showing the experimental protocol used). The heating thermograms obtained for the aged sample are shown in Figure 2. As predicted, the endothermic peak associated the enthalpy relaxation increased with aging time.

The enthalpy recovery (Δ*H_r_*) was determined by the integration of the total heat flow signal after subtracting the enthalpy calculated from an experiment without aging (under identical modulated conditions); Δ*H_r_* is represented as a function of the aging time in the right-hand-side inset of Figure 2. As it can be seen, at c.a. t_ag_ ~90 min, Δ*H_r_* reaches a constant value.

The kinetics of physical aging processes can be described using the Kohlrausch–Williams–Watts (KWW) empirical equation [29]:

(3) $$∆H_{r}\left(t\right)=∆H_{r}^{\infty }\left[1-exp\left(-{\left(\frac{t}{\tau_{KWW}}\right)}^{\beta }\right)\right],$$

where Δ*H*_r_ and $∆H_{r}^{\infty }$ are respectively the enthalpy of relaxation and the equilibrium enthalpy relaxation, *t* is the aging time, *τ_KWW_* is the relaxation time, and *β* is the coefficient describing the distribution of the relaxation times. The line included in the inset of Figure 2 with parameters $∆H_{r}^{\infty }$ = 1.67 J∙g^−1^, *τ_KWW_* = 1550 s and *β* = 0.8 represents a good fitting of the data, which corresponds to a broad distribution of the relaxation times attributable to some heterogeneity.

It is commonly accepted that by keeping the temperature under control and below certain limits, usually around 40 °C below the glass transition temperature, pharmaceutical manufacturers can consider that the drug remains efficient for the expected shelf life, often set at two years.

Proper packaging and humidity control are also critical factors for maintaining stability. In this study, the aging temperature selected, 5 °C, was chosen with the understanding that it is a typical operational temperature for storage conditions. Based on our results, it is recommended to store amorphous ACF at lower temperatures to minimize the impact of physical aging.

Apart from these results, it must be emphasized that at the end of the aging experiments, the sample was heated up to 170 °C and no melting signal was detected, demonstrating the high stability of amorphous ACF against crystallization.

### 2.2. FTIR Results

Since vibrational modes are sensitive to intermolecular interactions, FTIR analysis was used to gain deeper insights into how these interactions are influenced by modifications in the physical state. The FTIR spectrum of the ACF crystal phase sample showed well-defined bands associated with the specific vibrational modes of the crystal lattice. On the other hand, the infrared spectrum obtained from the designated amorphous phase, though it should be noted that the sample might not have been entirely amorphous, clearly indicated the absence of the long-range organization typical of a crystalline phase, as observed in the other sample. This is evident from the broad and less defined bands, which are characteristic of a more disordered molecular arrangement in this phase (see Figure S5 in the Supplementary Information).

As reported by Jelsch et al. [30], stabilization of the ACF tridimensional structure is achieved through the formation of several hydrogen bonds. Specifically, the carboxylic acid group interacts with two similar groups from two neighboring molecules, forming two strong C=O∙∙∙H-O bonds. Additionally, the C=O of the ester groups of two molecules participate in the dimer formation, and, at the same time, they are involved in the intramolecular interactions with the secondary amine group. The geometry of these directional intermolecular interactions is surely modified when the tridimensional long-term order of the crystalline state is destroyed during amorphization. Consequently, the infrared spectra were expected to reflect these changes, particularly in the vibrational modes associated with the carboxylic acid, ester and amine groups normally involved in this type of interaction.

**Figure 3:**
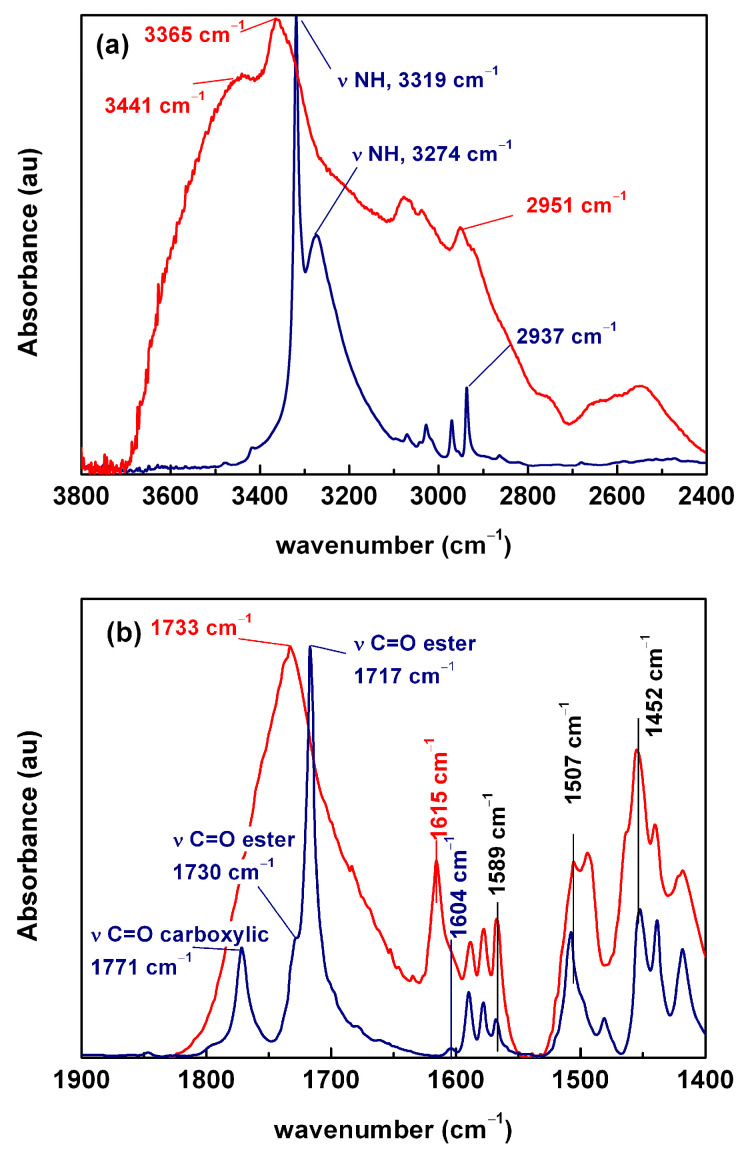
FTIR spectra of neat aceclofenac in crystalline (blue) and amorphous (red) forms at room temperature in the regions: (**a**) 3800–2400 cm^−1^ and (**b**) 1900–1400 cm^−1^. In both cases, spectra have been normalized to the corresponding maxima.

The complete FTIR spectra of both the crystalline and amorphous phases of ACF are presented in Figure S4 of the Supplementary Information. For clarity, two distinct regions of the spectra (3800–2400 cm^−1^ and 1900–1400 cm^−1^) are shown in Figure 3.

The 3800–2400 cm^−1^ region (Figure 3a) is dominated by the bands associated with the stretching modes of the -OH and -NH bonds, often presenting partial or total overlap. The crystalline ACF displays a sharp band of the amino group corresponding to the -NH stretching mode at 3319 cm^−1^ [1,8,11,18,21,31,32] and a lower intensity band, which may be attributed to the -OH stretching mode, at 3274 cm^−1^. The latter can be influenced by the presence of crystallization water as confirmed by the small intensity band at ~1603 cm^−1^ [33].

In the amorphous sample, the stretching -NH band shifted to higher wavenumbers and visibly broadened, as expected. These changes suggest that, in addition to the intramolecular –NH∙∙∙OH– bonds, which are dominant in the crystalline form, a variety of hydrogen bonds involving the amine and hydroxyl groups occur in the disordered amorphous state. Additionally, the shift of the maximum to higher wavenumbers also suggests a possible weaker interaction.

At approximately 2937 cm^−1^, several narrow, low-intensity bands were observed, which were attributed to the -OH stretching mode [11,34]. However, their position and shape were comparable to those expected for the stretching modes of the -CH, -CH_2_ and -CH_3_ groups [33] and were possibly better assigned to these vibrational modes.

In the region between 1900 and 1500 cm^−1^ of the infrared spectrum of aceclofenac (Figure 3b), it was expected that the following bands corresponding to various vibrations would be observed: carbonyl stretching (1700–1750 cm^−1^), aromatic ring stretching (~1600 cm^−1^), amine -NH bending (1600–1500 cm^−1^), and also the symmetric bending of the entrapped water in the solid structure (1650–1600 cm^−1^) [33].

The crystalline ACF spectrum was dominated by the higher intensity C=O stretching bands at 1717 cm^−1^ and 1771 cm^−1^, which can be associated respectively to the ester and the carboxylic acid groups. The presence of a shoulder around 1730 cm^−1^ in the C=O stretching mode of the ester group may indicate that this group probably participates concomitantly in intra- and intermolecular interactions (dimer formation) [30]; those with two hydrogen bonds may appear at lower wavenumbers (1717 cm^−1^), while those with C=O participating only in one would contribute to the band at a higher wavenumber (1730 cm^−1^).

The infrared spectrum of the ACF form, on the other hand, presented significant changes in the carbonyl stretching region. The carbonyl stretching band of the ester group overlapped with that of the acid group, giving rise to a very broad band. The apparent shift observed cannot be directly attributed to a band shift of the carbonyl modes, since a concomitant and opposite trend could result in a global shift to 1733 cm^−1^. In addition, the significant increase in the intensity of the C=O band below 1700 cm^−1^ can be linked to higher diversity and weaker strength of the hydrogen bonding interactions in the molecule.

In addition, the comparison of the spectra of the crystalline and amorphous phases suggests a few more observations, as follows:

(a) the C=C stretching modes (aromatic) located at 1589, 1508 and 1452 cm^−1^ did not shift significantly and remained well resolved;
(b) at 1604 cm^−1^, the FTIR spectrum of crystalline ACF showed a small shift that can be associated with vibrational modes related to the vibrational modes of carbon-carbon involved in multiple bonds (ν_CC_ + ν_CCC_) [35]. In contrast, for amorphous ACF, a distinct band appeared at 1615 cm^−1^, which may correspond to the same band shifted and with increased relative intensity. Notably, changes in this band were accompanied by variations in the OH stretching region. This correlation suggests the incorporation of water into the solid structure, consistent with the higher hydration capacity of the amorphous phase, which is thermodynamically less stable than its crystalline counterpart.

Taking into account that the intramolecular hydrogen bond between the amine and the oxygen in the ester group [30] was significantly weakened by amorphization (as also suggested by variations of the –OH band), the following two conclusions can be tentatively drawn: (i) in the crystalline phase, it can be made the assignment of dimeric interactions involving intramolecular hydrogen bonds to those centered at 1732 cm^−1^ and 1717 cm^−1^ (the latter according to the attribution made by Goud et al. [8]); and (ii) in the amorphous phase, we can rule out the existence of long-range interactions. These results show that some kind of short-distance organization remained in the amorphous state, which (as we will show later) can have a significant impact on molecular dynamics.

### 2.3. DRS Results

**Figure 4:**
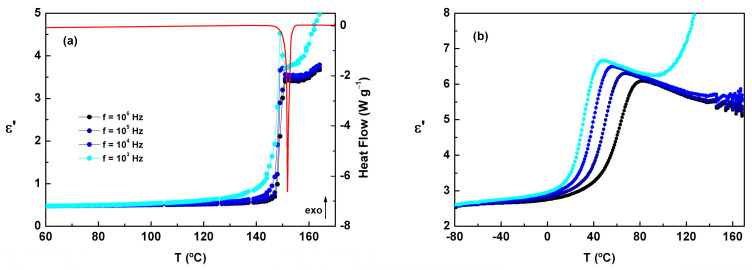
(**a**) (1) Blue and black lines (*ε*’ in the left axis): ε’(T) measured on heating the fresh sample at 5 °C∙min^−1^ up to 165 °C at different frequencies; (2) red line (heat flow on the right): DSC heating thermogram obtained under similar conditions. (**b**) *ε*’(T) measured on cooling; the correspondence between the color of the experiment and the frequency is the same as in (**a**).

By heating the as-received crystalline ACF sample up to 170 °C in the dielectric sample cell, melting was detected close to 150 °C by a sharp increase, independent of the frequency, in the *ε*’(T) trace (Figure 4a). Upon cooling from the liquid state (Figure 4b), *ε*’(T) increased until reaching a maximum that depends on frequency, and then it showed a pronounced decrease corresponding to the transition from the supercooled liquid to the glassy state.

According to the Kirkwood–Fröhlich model, which describes permanent dipoles immersed in a pure liquid considered as a continuous medium of dielectric constant *ε_∞_*, the initial increase in *ε*’(T) upon cooling can be described by the following relationship:

(4) $$∆\varepsilon =\left(\varepsilon_{s}-\varepsilon_{\infty }\right)=\frac{1}{3\varepsilon_{0}}g_{k}F\frac{\mu_{0}^{2}}{k_{B}T}\frac{N}{V}\;,$$

where *ε_0_* is the vacuum permittivity, *μ_0_* is the dipole moment of the moving unit in vacuum, T is the temperature, *k_B_* is the Boltzmann constant, *N*/*V* is the number of dipoles per unit of volume, *g_k_* is the Kirkwood correlation factor, *F* is the Onsager factor of the reaction field model, which takes values very close to unity, and *ε*_s_ and *ε*_∞_ are the limits of the real part of the dielectric permittivity at low and high frequencies, respectively. The *g_k_* value reflects intermolecular dipole-dipole interactions, where *g_k_* > 1 indicates a predominant parallel correlation, 0 < *g_k_* < 1 suggests an antiparallel correlation, and *g_k_* = 1 represents a random orientation.

Assuming *g_k_* as a constant, the prediction for ACF from Equation (4) is 12.5%, while the value obtained from the data is 8.8% (for f = 10^4^ Hz, T_1_ = 119.3 °C, and T_2_ = 71.3 °C; the temperatures provided are solely intended to exemplify the estimated calculation), which corresponds to *g_k_* < 1. This difference cannot be ascribed to recrystallization of the sample given that any signal associated with this phase transition was detected in ε*. The “loss” of the dipolar moment could be associated with the existence of some kind of association between ACF molecules, resulting in an overall reduction in the dipolar moment due to a dominant antiparallel configuration.

**Figure 5:**
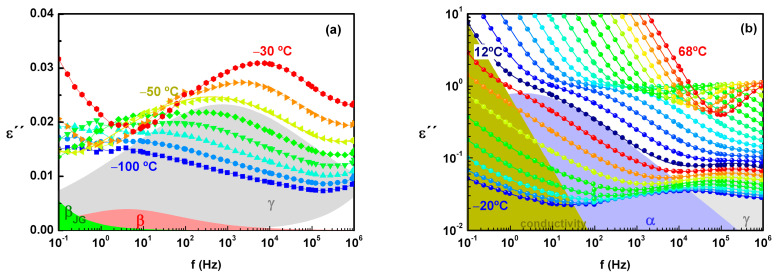
*ε*^″^(f) spectra collected on heating from the glassy state represented in different colors: (**a**) from −100 to −30 °C every 10 degrees and (**b**) from −20 to 68 °C every 4 degrees; for isothermal spectra collected at −50 °C (**a**) and 12 °C (**b**), individual and overall fitting functions are included as examples.

The isothermal *ε*^″^(f) spectra, collected on heating from the glassy state, displayed a broad and asymmetric peak at the lowest temperatures (Figure 5a). With further heating, the ionic conductivity became visible with the continuous increase in the low frequency side of the *ε*^″^ spectra, and, at higher temperatures, a narrow and intense peak became resolved from the conductivity tail (see Figure 5b). Considering that ACF is a non-ionic compound, the way conductivity and α relaxation appear in the frequency window, as well as how they behave with increasing temperature, suggests: (i) the dc conductivity is activated at temperatures close to or even below T_g_, i.e., it does not require total devitrification of the sample to activate long-term diffusion of charges, and (ii) above T_g_, the relaxational motions become faster as temperature increases and faster than the charge translation. These points are further discussed in the text.

From the *ε*^″^(f) spectra analysis, two secondary processes (identified as γ and β) were well resolved from the broad peak at lowest temperatures (see the spectrum at −50 °C in Figure 5a in which individual Havriliak–Negami fitting functions have been included). Above −30 °C, the β relaxation was overlapped by γ, resulting in an asymmetric broad peak. To achieve a good fit of the experimental results, it was necessary to consider the presence of a weak-intensity relaxation in the region between the β process and the conductivity tail.

To better resolve the α relaxation, spectra above −6 °C were analyzed in both *ε*^″^ and ($\varepsilon_{der}^{\prime \prime }=\left(-\pi /2\right)d\varepsilon^{\prime }/dln\omega$) representations [36,37]; the latter was used to validate the resolution and to extend the information about the α peak to lower temperatures since this procedure resulted in the elimination from the spectra of the pure dc contribution (see Figure S5 in the Supplementary Information as a an example of the proposed method). During the fitting procedure of $\varepsilon_{der}^{\prime \prime }$, the existence of an additional peak at higher temperatures and lower frequencies than the α one became clear; the corresponding shape fitting parameters (*α*_HN_ = *β*_HN_ = 1) indicated that it is a Debye peak, i.e., that it describes a narrow and symmetric distribution of relaxation times.

**Figure 6:**
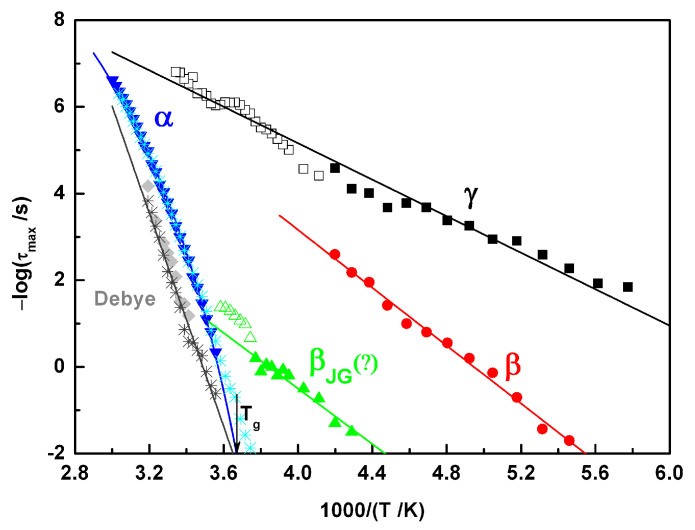
Relaxation map of amorphous aceclofenac obtained from the fitting of *ε^″^(f)* spectra: filled symbols for γ (black), β (red), β_JG_ (green), α (blue) and Debye (grey) indicate the data used for the corresponding Arrhenius or VFTH fitting. Asterisks correspond to relaxation times obtained from the fitting in $\varepsilon_{der}^{\prime \prime }\left(f\right)$ of α and Debye peaks respectively. The vertical arrow indicates the glass transition temperature value obtained from the extrapolation of the VFTH fitting line to *τ* = 100 s.

In addition to the shape parameters of the different mobilities (shown in Table 1), it is important to analyze their characteristic relaxation times, which are represented in Figure 6. For the sub-T_g_ β and γ secondary relaxations, the −log*τ* vs. 1/T plot followed a linear trend that can be well-described by the Arrhenius equation, $\tau \left(T\right)=\tau_{0}exp\left(E_{a}/RT\right)$, where *τ*_0_ is pre-exponential factor, *E_a_* is the activation energy, and R is the gas constant. The resultant fit parameters are included in Table 2. The low values of *E_a_* obtained for the β and γ relaxations lead us to consider that the underlying motions have an intramolecular nature. As noted from the direct observation of the spectra and also from the relaxation map, the tendency of both processes to merge with each other at higher temperatures seems very clear.

As the temperature increased, approaching T_g_, the dynamic behavior became more complex; the low-intensity process immediately after β (green triangles), although difficult to define clearly by fit analysis, undoubtedly underwent a change in the *τ*(T) trace. From the Arrhenius fitting, the activation energy obtained was 61.4 kJ∙mol^−1^, increasing significantly to higher values above ~−4 °C (see the open green triangles in Figure 6).

Regarding the main α process, the temperature dependence of the relaxation time (−log*τ* vs. 1/T) followed a non-linear trend. Considering only the *τ*(T) extracted from the analysis of ε^″^(f) (dark blue symbols in Figure 6), this behavior can be described by a VFTH equation [38,39,40]:

(5) $$\tau \left(T\right)=\tau_{0}exp\left(\frac{B}{T-T_{0}}\right)\;,$$

where *τ*_0_ and *B* are parameters and *T*_0_ is the Vogel temperature. The fitting parameters obtained are shown in Table 2. The value of T_g_ extracted from dielectric data can be estimated from the extrapolation of this fitting function to *τ* = 100 s [27], which corresponds to −0.6 °C for ACF (discrepancies are discussed in the Discussion Section). This T_g-DRS_ value is close to the temperature at which a change in the *E_a_* value was observed for the slower secondary process, which may suggest a correlation between these relaxations. On the other hand, while *τ*_α_(T) obtained from the $\varepsilon_{der}^{\prime \prime }\left(f\right)$ analysis (light blue symbols in Figure 6) coincided with that taken from *ε*^″^(f) at high temperatures, a clear deviation was observed at lower temperatures to a more linear trend. In this temperature region, the Debye peak whose relaxation times were almost equal to *τ*_α_(T) on approaching T_g_ became visible, indicating that the underlying mechanisms became active at the same temperatures. However, it seems important to note that unlike *τ*_α_(T), *τ*_Debye_(T) followed an Arrhenius tempe -rature dependence; the low value of the pre-exponential factor (*τ*_0_ = 10^−43^ s) points to a cross temperature with the *τ*_α_(T) trace at high temperatures. As was carried out for the DSC results, the activation energy can be estimated from *τ*(T):

(6) $$E_{a}\left(T_{g}\right)={\left[\frac{\partial ln\tau \left(T\right)}{\partial \left(\frac{1}{T}\right)}\right]}_{T=Tg}\;,$$

as well as the fragility index (Equation (2)), resulting in *E_a_*(*T_g_*) = 431 kJ∙mol^−1^ and *m* = 83, respectively.

#### Conductivity

**Figure 7:**
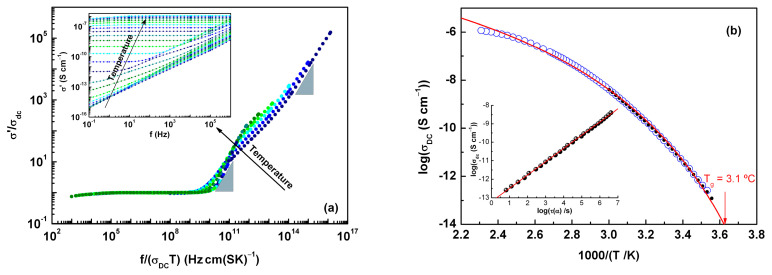
(**a**) Conductivity master curves generated by using Summerfield scaling for selected temperatures from 10 °C to 110 °C every 10 °C shown in different colors for better visualization; blue triangles indicate the slope equal to 1. Inset: *σ*’(f) spectra from −100 to 160 °C every 10 degrees. (**b**) *σ_dc_* values obtained from the plateau in *σ*’(f) as blue circles and from the fitting with the term $\sigma_{dc}/{\left(2\pi f\varepsilon_{0}\right)}^{n}$ (with *n* = 1) in *ε*^″^(f). The red line corresponds to the best fitting with a VFTH-type function for all data. Inset: log *σ_dc_* vs. log *τ_α_* for temperatures from 10 to 60 °C.

At lower temperatures, the real part of complex conductivity, *σ*’(f) (the inset of Figure 7a) showed a frequency-dependent regime, which changed as the temperature increased and approaches ~14 °C to the plateau corresponding to the direct current conductivity (*σ_dc_*) related to the random motion of the charge carriers. With further heating, a slight decrease was observed at lower frequencies, indicating the onset of electrode polarization. This effect, which involves charge accumulation at the electrode surface, was also seen as an increase in ε’ and a slope in log (*ε*^″^) lower than unity.

The *σ*’(f) spectra from 10 to 110 °C were normalized using ”Summerfield scaling” [41]. As observed in Figure 7, apart from the plateau corresponding to the *σ*_dc_, the sub diffusive regime (frequency dependent) does not collapse into a single master curve. The lack of a full overlap of the normalized spectra may be the result of the different contributions of more than one underlying relaxation. In fact, in this temperature range from 10 to 70 °C, the emergence of the intense α relaxation followed by a Debye peak was detected.

The values of *σ_dc_* taken directly from the plateau in the *σ*’(f) spectra are plotted as a function of inverse temperature in Figure 7b, where the non-Arrhenius temperature dependence is clear. The *σ_dc_* values obtained from the fitting with the term $\sigma_{dc}/{\left(2\pi f\varepsilon_{0}\right)}^{n}$ in Equation (8) has been also included as black symbols, showing very good agreement with the data taken from the *σ*’(f) representation. The solid line in Figure 7b represents the best-fitted curve of dc conductivity temperature variation using a VFTH-like equation. The obtained fitting parameters were log*σ*_0_ = −2.26 S∙cm^−1^, *B* = 1718 K and *T*_0_ = 212 K.

To evaluate the degree of coupling/decoupling of the translation of charges with the dynamics associated with the glass transition, in the temperature range in which α was also detected, the corresponding relaxation times, *τ*_α_, were represented in the log-log scale as a function of *σ_dc_* (log-log scale), evidencing a linear relation between both parameters (see inset in Figure 7b). When orientational relaxation (of the amorphous molecules) and translational motions (of charge carriers) are fully coupled, the slope expected in this representation is equal to unity, meaning that the relation $\sigma_{dc}\left(T\right)\propto 1/\tau_{\alpha }\left(T\right)$ is satisfied; otherwise, when both dynamics are decoupled, the description can be carried out by $\sigma_{dc}\left(T\right)\propto 1/{\tau_{\alpha }\left(T\right)}^{x}$, with the exponent 0 < x < 1 (see for instance ref. [42] and references therein). These two expressions are known as the Debye−Stokes−Einstein (DSE) and fractional DSE (FDSE) equations, respectively. For ACF, the value x = 0.72 was found, which is much lower than the values reported in the literature for other low-molecular-weight organic compounds (see Table 3). This fractional exponent was interpreted by Psurek et al. [43] in terms of the ratio between the activation volume required for each of the motions, $x=∆V_{\sigma }/∆V_{\alpha }$. A lower value of x indicates a more pronounced difference between the volume involved in the translation of small charges and that needed for the cooperative motion. Additionally, it was shown [43] that a larger molecule size enhances the decoupling between *σ* and *τ*_α_. However, from a comparison of the x values for several compounds with different molecular weights (see Table 3) and consequently their different molecular volumes, it seems clear that there must be other factors to explain the low value of x observed in ACF.

### 2.4. TSDC Results

#### 2.4.1. The *α*-Relaxation and Mobility Above T_g_

**Figure 8:**
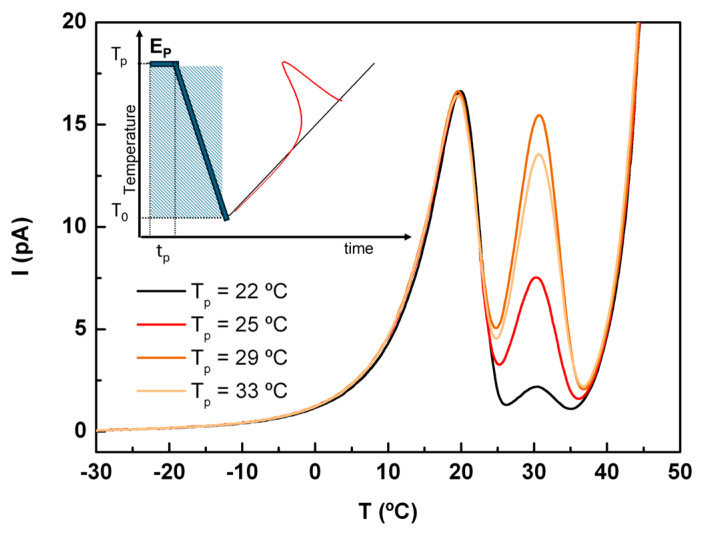
TSDC global results showing the α-relaxation and the relaxation above T_g_. The polarization temperatures were T_p_ = 22, 25, 29 and 33 °C; with the electric field applied, the freezing temperature and the heating rate were respectively 250 V∙mm^−1^, T_0_ = −40 °C, and 8 °C∙min^−1^. Inset: schematic representation of the global TSDC experiment; the electric field is active in the shaded area.

After ACF was amorphized in the TSDC sample cell (see the experimental section for details), four experiments were carried out in a global model using polarization temperatures of 22, 25, 29, and 33 °C (see Figure 8). The low temperature peak, with a maximum intensity at 19.6 °C, corresponds to the depolarization of the α mode. The fact that these peaks had identical intensity and shape at different polarization temperatures clearly indicates that all components of this mobility were activated at the lowest polarization temperature (i.e., at T_p_ = 20 °C). In contrast, the peak located at higher temperatures (T_max_ ~ 30.7 °C), hereafter referred to as the liquid-liquid (LL), showed an intensity that depends on T_p_, passing through a maximum for T_p_ = 29 °C. This behavior suggests that at a specific temperature (in this case, 29 °C) in the supercooled liquid regime, the polarization induced by the electric field was no longer retained. In other words, the thermal energy became sufficient to counteract the effect of the polarizing field, E_p_.

**Figure 9:**
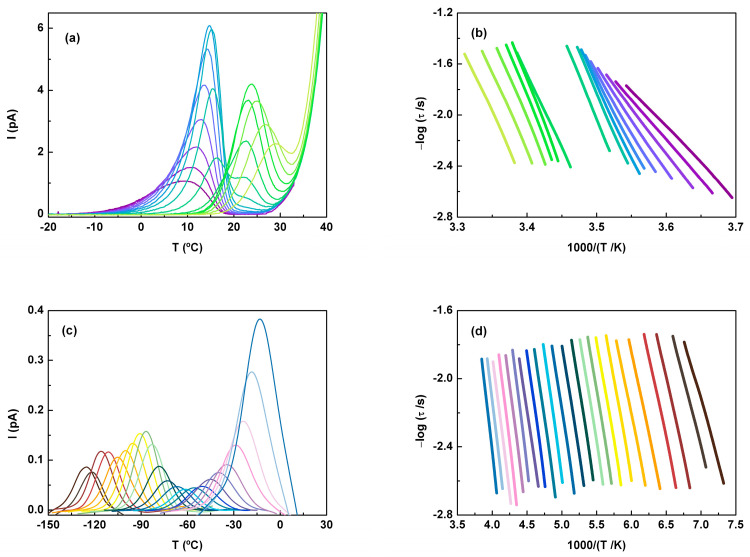
Partial polarization peaks displayed in different colors for: (**a**) T_p_ = [−2, 18] °C every 2 degrees with E_p_ = 250 V∙mm^−1^, and (**c**) T_p_ = [−130, −20] °C every 5 degrees with E_p_ = 350 V∙mm^−1^ (due to the low signal intensity, some smoothing was introduced). The other parameters are heating rate r = 6 °C∙min^−1^, ΔT = 2 °C. In (**b**,**d**), the logarithm of the relaxation times vs. the reciprocal of temperature for the peaks presented in the corresponding upper panels is displayed; the colors help to identify the relaxation times with the corresponding peaks in **b** and **c** figures.

The partial polarization method was applied to access the elementary (or partial) components of these global peaks. As shown in Figure 9, although the two maxima are close to each other, this procedure, with a polarization window of 2 degrees (ΔT = 2 °C), allows us to distinguish between the two modes. Note that the conductivity tail at higher temperatures does not significantly interfere with the polarization peaks, allowing a proper mathematical analysis of them.

From two sets of PP windows analyzed with T_p_ varying from −2 to 30 °C, the glass transition temperature estimated from the position of the maximum intensity of the PP peak was 14.5 ± 0.2 °C. This value is close to the onset determined by DSC (T_g-on_ = 12.6 °C) but is clearly different from that estimated by DRS (T_g_ (*τ* = 100 s) = −0.6 °C). Considering the sample cell configuration of DRS, one might suspect the possibility of some water absorption during the measurements. However, this was ruled out due to the pre-heating up to 170 °C (for ACF melting) and the fact that the values of the dielectric properties did not suggest the presence of water. Based on the good agreement obtained between the results of the three techniques for other materials (see for instance [49], where T_g_ values for more than twenty low-molecular-weight compounds were compared), the differences observed here, mainly between T_g_ from DRS and T_g_ by TSDC or DSC, lead us to assume that this is an intrinsic feature of ACF, which is discussed in next section.

Regarding the relaxation times (estimated as described in the experimental section; an example can be found in the Supplementary Information), the α components exhibited a slight curvature, as shown in Figure 9. These components tended to merge as the temperature approached T_g_, a behavior commonly observed in many glass-forming materials. This behavior can be described by the William–Landel–Ferry equation [50]:

(7) $$\tau \left(T\right)=\tau_{0v}exp\left(\frac{1}{\alpha_{f}\left(T-T_{\infty }\right)}\right)\;,$$

where *T*_∞_ is a temperature whose determination allows a linearization of $log\left(T\right)=f\left(1000/\left(T-T_{\infty }\right)\right)$, *α_f_* is the free volume dilatation coefficient, and *τ*_0*v*_ is the pre-exponential factor. The fragility index estimated by TSDC from the partial polarization peak of higher intensity was 54.

**Figure 10:**
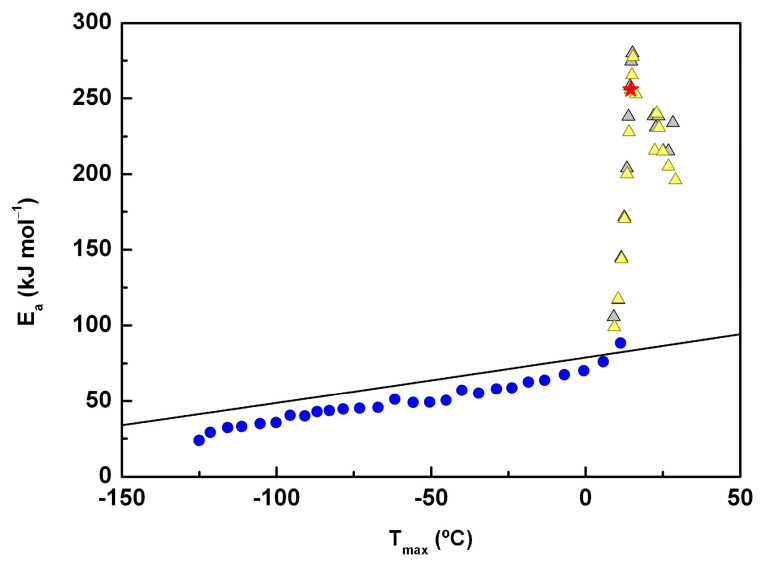
Relaxation map of ACF displaying the activation energy, *E_a_*, of the partial polarization components of the TSDC spectrum as a function of the temperature of maximum intensity, T_M_, of the corresponding current peak. Results from three series of partial polarization peaks with T_P_ varying from −130 to 30 °C are displayed (each series is identified with a symbol and color). *E_a_* obtained for the PP peak corresponding to the T_g-TSDC_ is represented by a red star. The black line corresponds to the zero-entropy line (ZEL).

On the other hand, by analyzing the mobility above the glass transition temperature, we found T_max_ = 23.6 °C for the elemental peak with the highest intensity, which was obtained in the partial polarization experiment with T_p_ = 22 °C. Note that the dependence on the inverse of the temperature of relaxation times is linear in the relaxation map (Figure 9b). From the fitting using an Arrhenius-type function, the corresponding activation energies were estimated. These values are represented as a function of the temperature of the maximum intensity in Figure 10. The black line in this figure is the so-called zero activation entropy line (ZEL), which depicts the behavior of the mobility modes with Arrhenius pre-factors close to the Debye value *τ_0_* ∼10^−13^ s, representative of the local and non-cooperative thermally activated processes. For a more detailed explanation of how to obtain the ZEL, see [51].

As shown in Figure 10, the *E_a_* values for both the α and LL components are clearly higher than those predicted by the ZEL, confirming the cooperative/non-local nature of both mechanisms.

**Figure 11:**
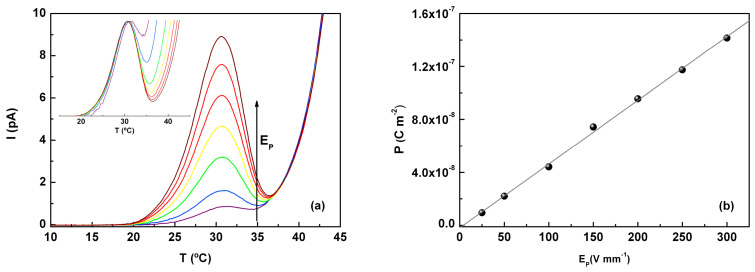
(**a**) Effect of the electric field strength on the polarization of the partial polarization component of the liquid-liquid mode obtained with T_p_ = 29 °C, T_0_ = −20 °C, T_end_ = 45 °C, r = 6 °C∙min^−1^, ΔT = 2 °C; E_p_ = 25, 50, 100, 150, 200, 250, and 300 V∙mm^−1^, each of TSDC curves identified with a color. Inset: I/I_max_ vs. T (curve normalization). (**b**) Polarization of each depolarization peaks in (**a**) vs. the corresponding applied electric field (E_p_); the solid line describes the linear correlation between the two quantities.

To better understand the nature of this mobility above T_g_, a set of experiments were conducted by varying the intensity of the polarizing electric field, E_p_, with other experimental parameters kept constant (see also the TSDC section in Suplementary Information). As shown in Figure 11a, the intensity of the peaks increased as E_p_ increased, while the temperature of the maximum remained nearly constant, 30.8 ± 0.2 °C (for E_p_ > 50 V∙mm^−1^, T_max_ = 30.7 ± 0.1 °C). Additionally, the polarization calculated for each peak varied linearly with E_p_ (Figure 11b), as expected for a purely dipolar response. The activation energy, estimated from the temperature dependence of the relaxation times (Arrhenius type) remained nearly constant, suggesting that the molecular motions involved did not change with the intensity of the electric field; in other words, no additional mechanisms were activated by the action of the electric field, E_p_. This conclusion is further supported by the normalized curves (inset in Figure 11a), which show that despite the proximity to the conductivity tail, the shape of the curves remains almost unchanged.

The origin of the depolarization peaks observed above the α relaxation has not yet been rigorously identified, although they are commonly observed. In polymers, for example, peaks above T_g_ are often attributed to charge detrapping (*ρ* peak) [52,53]. A similar phenomenon could occur in a low-molecular-weight organic compound like aceclofenac, which is non-ionic in nature. However, the high reproducibility of this occurrence makes it difficult to attribute the peaks solely to impurities.

On the other hand, in addition to the high molecular mobility resulting from the decrease in viscosity above T_g_, which makes it difficult to retain polarization, the presence of dipolar relaxations should also be considered. Returning to the case of polymers, if the dipole moment is located in the main chain, the corresponding peaks are associated with the normal mode [52,54]. In other cases, the occurrence of a liquid-to-liquid transition has been suggested [53,55]. In liquid crystals for instance [56], the peaks above T_g_ have been attributed to “molecular reorientations around the short axis of the molecule”, while in alcohols [57], they have been linked to the formation of supramolecular structures via hydrogen bonds [58]. In amorphous aceclofenac (like in other organic compounds such as brucine [47]), the well-defined shape and reproducibility of the peaks above T_g_ lead us to hypothesize that without completely discarding the ionic species motion, some genuine type of dipolar rearrangements must be involved, pointing to a liquid-liquid (LL) transition.

#### 2.4.2. Relaxation in the Sub-T_g_ Region

The resolution by DRS of several relaxations below T_g_ also led us to analyze the depolarization thermo-currents in this temperature region in more detail. For this purpose, the partial polarization (PP) window procedure was extended to polarization temperatures as low as −120 °C (Figure 9c).

The plot of the maximum intensity (I_max_) vs. polarization temperature (T_p_) showed a maximum at T_p_ = −90 °C (T_max_ = −86.8 °C), suggesting that more than one relaxation mode was covered by this large temperature window. The corresponding relaxation times are shown in the lower panel of Figure 9d, where we can clearly see a temperature depen- dence that follows Arrhenius’ law.

The pre-exponential factors estimated for T_p_ value’s down to −15 °C took values of the order of 10^−12^ s, and the activation energies fell very close to the zero-entropy line (ZEL) (see Figure 10), meaning that they are representative of local relaxations for which the activation enthalpy (or activation energy) depends slightly on T_m_ in an approximately li near way [59]. On the other hand, for T_p_ values varying from −10 to 5 °C inclusively, although the *E_a_* remained at the ZEL, the *τ_0_* values estimated started to decrease, pointing to a change in the underlying molecular motions, as also suggested by I_max_ temperature variation. On the other hand, for T_p_ values between −10 and 5 °C (inclusive), although *E_a_* remained at the zero-entropy line, the estimated values for *τ_0_* began to decrease. This suggests a change in the underlying molecular motions, which is further supported by the variation of I_max_ with temperature.

With regard to the origin of secondary relaxations, it is customary to classify them into two distinct groups, as follows: (a) those that correspond to rotations of a small part of the molecule, and (b) those that involve the movement of the molecule as a whole; these mobilities are usually referred to as Johari–Goldstein (JG) relaxations in honor of the authors who first suggested this distinction [60,61].

**Figure 12:**
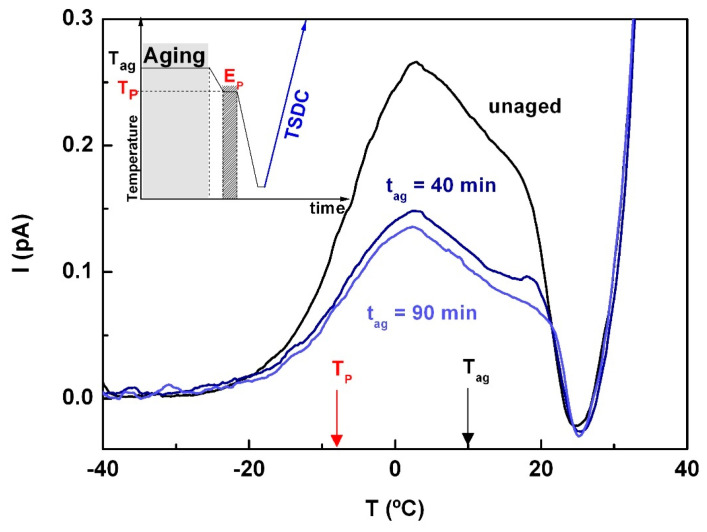
Partial polarization experiments conducted at T_p_ = −8 °C (E_p_ = 300 V∙mm^−1^, ΔT = 2 °C, r = 6 °C∙min^−1^, T_0_ = −40 °C, T_end_ = 40 °C) for unaged (black line) and aged samples at T_aging_ = 10 °C (dark blue line) over 40 and 90 min (light blue line). The inset represents the experimental procedure followed.

In TSDC, the sensitivity of different secondary relaxations to physical aging has been used as a probe to identify the JG components [62]. In this context, an aging experiment was performed in which the sample was kept at 10 °C for various periods of time, followed by polarization at T_p_ = −8 °C (the remaining experimental parameters are those used in the series of the partial polarization window in the sub-vitreous region). This experimental protocol is schematized in the inset of Figure 12. As shown in the main figure, a significant decrease in peak intensity was observed with aging, suggesting that this relaxation corresponds to a JG relaxation.

## 3. Discussion

The dynamic behavior associated with the glass transition of ACF can be studied using DRS, TSDC, and TMDSC due to the high stability of its amorphous form, which is obtained by cooling from melt. Good agreement was found for the fragility index, indicating that ACF is a moderately fragile glass former. However, significant differences were observed between the values of the glass transition temperature (T_g_) found through the different techniques—those obtained by TMDSC and TSDC were reasonably in agreement with each other but differed from those obtained by DRS.

The two following factors may explain the discrepancies in the T_g_ values: (a) the dc conductivity, which appeared to be active just below the devitrification temperature of the sample when heated from the glassy state, and (b) the presence of a post-T_g_ depolarization peak, which was clearly observed in TSDC.

The activation of the charge translation just below the dielectric T_g_ suggests that dc conductivity is not initially related to the main relaxation process but is more likely associated with secondary relaxations. In a vitrified matrix, where, as we will explain later, zones of different densities can coexist as a result of the amorphization process, charge translation can occur across the boundaries between regions, which resemble channels. This translation may be assisted by small (localized) rotations of molecules in adjacent regions, potentially aiding in the ‘anticipation’ of sample devitrification. These hypotheses would need further experimentation and likely the application of various techniques for validation.

With further heating above T_g_, as the supercooled liquid became less viscous, the dynamics associated with the glass transition accelerated, leading to a clear decoupling from charge motions. This was confirmed by the exponent value of the DSE relation, *x* = 0.72. Given that charge motions became active just below T_g_, one might expect a pronounced accumulation of charge at the electrode surface. However, at higher temperatures, far from the glass transition, it is interesting to note that the magnitude and extent of electrode polarization were relatively small (as seen on the low-frequency side in Figure 7a). Two possible explanations for this observation are: (i) a reduced number of charges reaching the electrode surface or (ii) the rate of charge arrival at the electrode surface allowing continuous discharging.

Additionally, the pronounced decoupling between relaxation and translation motion may be due to the presence of heterogeneity in the supercooled liquid [63].

As a consequence of this heterogeneity, an additional process may arise on the low-frequency side of the α relaxation, which is masked by conductivity in the DRS *ε*^″^(f) spectra. However, this process can be resolved in the TSDC signal as a post-T_g_ peak. In the case of ACF, shown in Figure 8, the separation between the peak associated with the main relaxation and the post-T_g_ signal is clearly visible in contrast to observations in other low-molecular-weight compounds such as carvedilol or salsalate [64,65], where the peaks are partially overlapped. Furthermore, the linear response of this peak to the polarizing electric field indicates a high stability of the heterogeneity “promoted” during the formation of the amorphous form. In other words, the degree of heterogeneity did not vary significantly once the liquid is supercooled.

Heterogeneity might be caused by the presence of larger or smaller aggregates, as seen in other compounds like mannitol [66,67] in which two different glass transitions have been observed or ibuprofen [68], which also contains a carboxylic acid group. This group has been linked to the formation of dimers and trimers held together by hydrogen bonds. In fact, the decrease in Δ*ε*(T) compared to the Frohlich-Kirkwood model prediction suggests a preferential antiparallel alignment, which supports this idea. Additionally, differences in FTIR spectra between the amorphous and crystalline samples show that the broadness of several bands points to a variety of hydrogen bonds in the amorphous sample, likely helping to stabilize these aggregates.

Regarding the mobility of ACF in the deep glassy state, TSDC experiments showed that the PP peaks, even with kinetic parameters above the zero-entropy line (i.e., not involving entropy changes, as seen in Figure 12 for T_p_ = −8 °C), were sensitive to physical aging. This characteristic has previously been used to identify JG relaxation [69,70,71,72,73]. In DRS, a poorly resolved peak may be associated with this relaxation, but only a noticeable change in the activation energy near the dielectric T_g_ supports this possibility. No significant changes in Δ*ε* or the estimated relaxation time from the CM model contribute to this identification.

At lower temperatures, a broad relaxation observed in the global TSDC data was deconvoluted into multiple components, showing a wide distribution of activation energies and pre-exponential factors. Using the equivalent frequency technique from TSDC (f ≈ 10^−3^ Hz, *τ* = 1.59 × 10^−2^ s), the Arrhenius fit for the β relaxation determined by DRS (*E_a_*(β) = 64 kJ∙mol^−1^ and *τ*_0_(β) = 3.04 × 10^−17^ s) estimates a temperature near −95 °C, which matches well with values obtained from TSDC and falls within the ZEL, as shown in Figure 9c.

For the γ relaxation, the same calculations placed the TSDC peak temperature near −140 °C, which is not accessible with the available equipment and cooling agent.

Even though dielectric techniques cannot directly pinpoint the molecular cause of any secondary relaxation, we can still make some informed guesses. The γ relaxation, based on ACF’s chemical structure, might be linked to ‘molecular fluctuations of carboxylic groups’ just like with ibuprofen [68], where the γ mode shows similar activation parameters (*E_a_* = 30.5 kJ∙mol^−1^ and *τ*_0_ = (6 ± 4) × 10^−16^ s). Similarly, gemfibrozil, which has the same functional group, also shows a quick secondary relaxation [74], which could be compatible with this assignment. It is also important to mention that in both compounds, stable dimers (or even trimers, etc.) involving the carbonyl groups have been observed.

**Figure 13:**
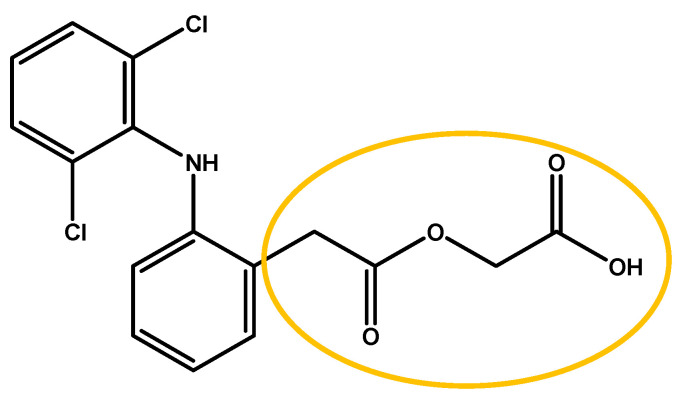
Chemical structure of aceclofenac emphasizing the groups acid and amine.

Concerning the β relaxation, we can guess that the motion involves a part of the molecule indicated in Figure 13. To validate this assumption, data from ss-NMR analysis and MD simulations would be very valuable.

## 4. Experimental

### 4.1. Materials

Aceclofenac, molecular weight of 354.2 g∙mol^−1^, whose chemical structure is presented in Figure 13, was supplied by TCI (CAS 89796-99-6, purity (HPLC) 99.5%; batch 70S5F).

### 4.2. Differential Scanning Calorimetry (DSC)

DSC analyses were carried out using DSC2920 and DSC Q2000 from TA Instruments (New Castle, DE, USA) under helium or nitrogen gas flows of 30 and 50 mL∙min^−1^, respectively.

Standard indium was used to calibrate the DSC apparatus, ensuring accurate measurements of the melting temperature and melting enthalpy.

Samples of ACF with masses from 3 to 8 mg were introduced in aluminum pans with pinholes to facilitate the evaporation of any residual water. For conventional DSC measurements, the sample was equilibrated at 20 °C before starting the heating at 5 °C∙min^−1^ up to 170 °C to induce the melting of ACF. Next, the sample was cooled down to −50 °C at 10 °C∙min^−1^ and heated up again to 170 °C at 5 °C∙min^−1^.

To investigate the kinetics of physical aging, the melted sample was cooled down to −50 °C, heated up to 5 °C (both steps at 20 °C∙min^−1^), and isothermally held for a specific aging time before cooling again. Next, it was heated under modulated DSC to 80 °C at 5 °C∙min^−1^, with an amplitude of 0.66 °C and a period of 60 s. The DSC data were analyzed using TA Universal Analysis Q2000.

### 4.3. Fourier Transform Infrared Spectroscopy (FTIR)

For infrared analysis, the samples were appropriately diluted with KBr to form thin, transparent pellets. The transmission infrared Fourier transform spectra were recorded using a Bruker Alpha T spectrometer (Universal Sampling Module). The spectra were scanned over the 4000–400 cm^−1^ range, with a resolution of 4 cm^−1^, based on 32 scans and using pure KBr as the background. The raw data file was imported into OPUS Spectroscopy software, version 7.5, for viewing, smoothing, and trimming of the spectra.

### 4.4. Dielectric Relaxation Spectroscopy (DRS)

The Alpha-N impedance analyzer from Novocontrol Technologies GmbH was used, covering the frequency range from 10^−1^ Hz to 1 MHz. Approximately 10 mg of sample was slightly compressed between two gold-plated electrodes (upper electrode, 10 mm diameter) joined to two silica spacers to maintain a constant distance between electrodes after melting. The sample capacitor was inserted in the BDS 1200 sample holder and mounted on a BDS 1100 cryostat, and the sample temperature (±0.5 °C) was controlled by a Quatro Cryosystem from Novocontrol.

Isothermal ε*(f) spectra were measured by increasing the sample temperature from −100 °C to 160 °C in a stepwise manner, with steps of 5 or 2 °C (depending on the temperature range: from −100 °C to −20 °C and from 100 °C to 160 °C every 5 degrees and between −20 and 100 °C every 2 degrees).

#### DRS Data Analysis

The imaginary component of the ε*(ω) isothermal spectra was analyzed by using a sum of several Havriliak–Negami (HN) [75] functions that read:

(8) $$\varepsilon^{*}\left(\omega \right)=\epsilon_{\infty }+\sum_{j}\frac{∆\varepsilon_{j}}{{\left[1+{\left(i\omega \tau_{HNj}\right)}^{\alpha_{HNj}}\right]}^{\beta_{HNj}}}-i\frac{\sigma }{\varepsilon_{0}{\left(2\pi f\right)}^{n}}\;,$$

where *j* is the index over which the relaxation processes are summed, Δ*ε = ε_s_ − ε_∞_* is the dielectric strength (the difference between the real permittivity values at the low and high frequency limits), *τ_HN_* is the characteristic HN relaxation time, and *α_HN_* and *β_HN_* are fractional parameters (0 < *α_HN_* < 1 and 0 < *α_HN_ β_HN_* < 1) describing a model-independent rela-xation time, *τ*_max_ = (2π*f*_max_)^−1^, which was calculated according to

(9) $$\tau_{max}=\tau_{HN}{\left[\frac{sin\left(\frac{\alpha_{HN}\beta_{HN}\pi }{2+2\beta_{HN}}\right)}{sin\left(\frac{\alpha_{HN}\pi }{2+2\beta_{HN}}\right)}\right]}^{1/\alpha_{HN}}\;.$$

The term $-i\sigma /\left({\left(2\pi f\right)}^{n}\varepsilon_{0}\right)$ was included in Equation (8) to take into account the direct current (dc) conductivity contribution on the low frequency side—*σ* is related to the dc conductivity, and *n* describes the broadening of the relaxation time distribution for dc conductivity (See chapter 3 in ref. [76] for more details).

### 4.5. Thermostimulated Depolarization Currents (TSDC)

TSDC has a low equivalent frequency (~2 × 10^−3^ Hz), offering high resolution and sensitivity to slow molecular motions (1–3000 s). Since relaxation times depend on temperature and increase as it decreases, cooling the sample can extend the relaxation time beyond the experiment’s timescale (freezing the process). In addition, TSDC measurements allow a high degree of flexibility in the analysis of relaxations since it is possible to examine specific fragments of the relaxation by optimizing the polarization step. Furthermore, if the sample is physically aged before the polarization step, it is possible to study the material in different structural states [77].

Two types of experiments are typically used in TSDC, including global experiments with a wide polarization window and partial polarization (PP) experiments with a narrow window. The PP procedure, also known as fractional polarization, is employed to examine specific regions of the TSDC spectrum. Two key parameters are the polarization temperature, T_P_, when the electric field is applied and T_p_’ (<T_P_) when the field is turned off, with the difference, ΔT = T_p_’ − T_P_, defining the polarization window width. A wide window results in a complex set of motional modes, while a narrow window probes more closely distributed relaxation modes. In the limit of an extremely narrow window, the depolarization current peak corresponds to a single relaxation mode. In this work, we used a two-degree wide polarization window assuming that it would isolate single relaxation processes, as similar results were observed with windows ranging from 0.5 to 2 degrees.

The analysis of TSDC data, specifically the partial polarization current peaks, entailed calculating the temperature-dependent relaxation time, τ(T), for the corresponding motional mode [78]. The mathematical methodology for analyzing partial polarization peaks is outlined in the appendix of reference [79].

## 5. Conclusions

In this work, the molecular mobility of amorphous aceclofenac obtained by cooling after melting was investigated. Although the glass transition temperature determined by DRS was considerably lower than that estimated by DSC or TSDC (from T_g-DRS_ = −0.6 °C to T_g-DSC_ = 12.6 °C and T_g-TSDC_ = 14.5 °C), the fragility index obtained by three techniques allows classification of ACF as moderately fragile (in the window *m*_TSDC_ = 54, *m*_DSC_ = 64 and *m*_DRS_ = 83).

In the deep glassy state, two secondary relaxations, γ and β, were resolved from the DRS spectra. The β relaxation is consistent with a mode also detected by TSDC at lower temperatures (~−95 °C). The activation parameters suggest that both relaxations corres-pond to localized motions within the ACF molecule without involving entropy changes.

Aging experiments conducted by TSDC, selecting a partial polarization peak with T_p_ just below T_g_ (where the activation energy lies on the zero-entropy line), show a clear reduction in intensity. This suggests a non-local nature of the relaxation, consistent with a JG assignment. Additionally, aging tests conducted at T_ag_ = 5 °C (approximately seven degrees below T_g_) showed a marked increase in the enthalpy of recovery when measured by TM-DSC. This indicates that the temperature is insufficient to stabilize the amorphous state.

In the supercooled liquid state, just above the α relaxation, TSDC revealed a peak whose intensity responds linearly to the intensity of the applied electric field. In this temperature range, the DRS spectra were characterized by high conductivity; the dc conductivity values correlated with the alpha relaxation times using the fractional Debye–Stokes–Einstein (FSDE) equation with the exponent x = 0.72. When the derivative analysis of *ε*’ was performed, an additional Debye peak was resolved. This peak in the supercooled liquid is attributed to the formation of aggregates due to intermolecular hydrogen bonding. This finding is consistent with the differences observed in the FTIR spectra of both the crystalline and amorphous phases.

In summary, this study shows that the dynamic behavior of amorphous aceclofenac is quite complex. This complexity could provide valuable insights for the development of ongoing and future pharmaceutical formulations.

## Figures and Tables

**Table 1:** Shape Havriliak–Negami fitting parameters.

Relaxation	α_HN_	β_HN_	T Range (°C)
γ	0.27 ± 0.03	1	[−100, 8]
β	0.46 ± 0.06	1	[−90, −35]
β_JG_	0.50 ± 0.11	1	[−50, 6]
α	0.46 at 8 °C, to 0.77 at 70 °C	1	[8, 60]
Debye	1	1	[20, 40]

**Table 2:** Fitting parameters of the Arrhenius and VFT equations for secondary and α relaxations detected in amorphous ACF.

Relaxation	−log (*τ_0_* (s))	*E*_a_ (kJ∙mol^−1^)	
γ	13.6 ± 0.2	40.3	
β	16.5 ± 0.5	63.9	
β_JG_	12.3 ± 1	61.4	
Debye	43.0 ± 1.4	236.1	
	**−log (*τ_0_* (s))**	**B (K)**	**T_0_ (K)**
α *	14.0 ± 0.5	1946 ± 189	219 ± 4

(*) VFTH parameters considering only *τ_α_*(T) taken from the *ε*^″^(f) analysis.

**Table 3:** The values of the exponent in the FSDE equation for some examples amorphous pharmaceuticals.

Compound	x Exponent	Mw (g∙mol^−1^)	Reference
Aceclofenac	0.72	354.2	
1PODGE	0.75	456.6	[43]
S-Flurbiprofen	0.76	244.3	[44]
Telmisartan	0.82	514.6	[45]
Prilocaine	0.92	220.3	[46]
Brucine	0.93	394.4	[47]
Fenofibrate	1	360.8	[48]

## Data Availability

Data will be made available on request.

## Supplementary Materials

The following supporting information can be downloaded at https://www.mdpi.com/article/10.3390/molecules30030681/s1: Figure S1. Melting and subsequent cooling thermograms of ACF; Figure S2. Heating thermogram of amorphous ACF; Figure S3. Microstructure of ACF formed during heating amorphous ACF; Figure S4. FTIR spectra of crystalline and amorphous ACF samples; Figure S5. Isothermal spectrum at 28 °C of amorphous ACF representing *ε^″^* and *ε′_der_*, and including the individual HN fitting functions and the overall fitted curve; Figure S6. TSDC global results of amorphous ACF obtained varying T_0_; Table S1. Real heating rates and the temperature onset of the glass transition of thermograms depicted in Figure 2.

## References

[1] Sheelarani B., Raj E.P., Joshi R.G., Dash S. (2022). Effect of drug aceclofenac on physico-chemical properties of mixed micellar systems. SN Appl. Sci..

[2] Iolascon G., Giménez S., Mogyorósi D. (2021). A Review of Aceclofenac: Analgesic and anti-inflammatory effects on musculoskeletal disorders. J. Pain Res..

[3] Brogden R.N., Wiseman L.R. (1996). Aceclofenac: A review of its pharmacodynamic properties and therapeutic potential in the treatment of rheumatic disorders and in pain management. Drugs.

[4] Polymorph I of ACF Was Approved in the EU by the EMA in 1990. https://www.ema.europa.eu/en/medicines/what-we-publish-medicines-and-when#human-medicines-38645.

[5] Ranpise N.S., Kulkarni N.S., Mair P.D., Ranade A.N. (2010). Improvement of water solubility and in vitro dissolution rate of aceclofenac by complexation with β-cyclodextrin and hydroxypropyl-β-cyclodextrin. Pharm. Develop. Technol..

[6] Soni T., Nagda C., Gandhi T., Chotai N.P. (2008). Development of discriminating method for dissolution of aceclofenac marketed formulations. Dissolution Technol..

[7] Shete A.S., Yadav A.V., Doijad R.C. (2022). Screening of aceclofenac for cocrystallization with nicotinamide: Theoretical and practical perspective. Indian J. Pharm. Sci..

[8] Goud N.R., Suresh K., Nangia A. (2013). Solubility and stability advantage of aceclofenac salts. Cryst. Growth Des..

[9] Shakeel F., Baboota S., Ahuja A., Ali J., Aqil M., Shafiq S. (2007). Nanoemulsions as vehicles for transdermal delivery of aceclofenac. AAPS PharmSciTech.

[10] SSakhare, Shinde S.D., Yadav A.V., Shete A.S. (2021). Studies on formulation and evaluation of eudragit RS PO based nanoparticulate system of aceclofenac for ocular delivery. Indian J. Pharm. Educ. Res..

[11] Patnaik S., Aditha S.K., Rattan T., Kamisetti V. (2015). Aceclofenac-Soluplus® nanocomposites for Increased Bioavailability. Soft Nanosci. Lett..

[12] Katara R., Sachdeva S., Majumdar D.K. (2017). Enhancement of ocular efficacy of aceclofenac using biodegradable PLGA nanoparticles: Formulation and characterization. Drug Deliv. Transl. Res..

[13] Thamer A.K., Abood A.N. (2021). Preparation and in vitro characterization of aceclofenac nanosuspension (ACNS) for enhancement of percutaneous absorption using hydrogel dosage form. Iraqi J. Pharm. Sci..

[14] Patil L.D., Verma U., Patil U.D., Naik J.B., Narkhede J.S. (2019). Inclusion of Aceclofenac in Mesoporous Silica Nanoparticles: Drug Release Study and Statistical Optimization of Encapsulation Efficiency by Response Surface Methodology. Mater. Technol..

[15] Jani R., Jani K., Setty C.M., Patel D. (2009). Preparation and Evaluation of Solid Dispersions of Aceclofenac. Int. J. Pharm. Sci. Drug Res..

[16] Yu L. (2001). Amorphous pharmaceutical solids: Preparation, characterization and stabilization. Adv. Drug Deliv. Rev..

[17] Hancock B.C., Zografi G. (1997). Characteristics and significance of the amorphous state in pharmaceutical systems. J. Pharm. Sci..

[18] Chishti N.A.H., Pathan I.B., Dehghan M.H.G., Bairagi S.M. (2024). Design and development of immediate release pellets formulation containing coamorphous mixture of aceclofenac: In-vitro and in-vivo Study. J. Pharm. Innov..

[19] Natarajan R., Komala G., Ramcy T.R., Polineni S., Mohan S. (2014). Dissolution enhancement of aceclofenac solid dispersion prepared with hydrophilic carriers by solvent evaporation method. Int. J. Res. Pharm. Chem..

[20] Maulvi F.A., Dalwadi S.J., Thakkar V.T., Soni T.G., Gohel M.C., Gandhi T.R. (2011). Improvement of dissolution rate of aceclofenac by solid dispersion technique. Powder Technol..

[21] Sipos E., Kósa N., Kazsoki A., Szabó Z.-I., Zelkó R. (2019). Formulation and characterization of aceclofenac-loaded nanofiber based orally dissolving webs. Pharmaceutics.

[22] Baird J.A., Van Eerdenbrugh B., Taylor L.S. (2010). A classification system to assess the crystallization tendency of organic molecules from undercooled melts. J. Pharm. Sci..

[23] Moynihan C.T., Easteal A.J., Wilder J., Tucker J. (1974). Dependence of the glass transition temperature on heating and cooling rate. J. Phys. Chem..

[24] Sellarès J., Cañadas J.C., Diego J.A., Mudarra M., Belana J. (2005). Application of the Tool–Narayanaswamy–Moynihan model to the study of the α relaxation by thermally stimulated depolarization currents. arXiv.

[25] Weyer S., Huth H., Schick C. (2005). Application of an extended Tool–Narayanaswamy–Moynihan model. Part 2. Frequency and cooling rate dependence of glass transition from temperature modulated DSC. Polymer.

[26] Angell C.A. (1991). Relaxation in liquids, polymers and plastic crystals: Strong/fragile patterns and problems. J. Non-Cryst. Solids.

[27] Böhmer R., Ngai K.L., Angell C.A., Plazek D.J. (1993). Nonexponential relaxations in strong and fragile glass formers. J. Chem. Phys..

[28] Drogón A., Skotnicki M., Skotnicka A., Pyda M. (2020). Physical ageing of amorphous indapamide characterised by diferential scanning calorimetry. Pharmaceutics.

[29] Williams G., Watts D.C. (1970). Non-symmetrical dielectric relaxation behaviour arising from a simple empirical decay function. Trans. Faraday Soc..

[30] Jelsch C., Devi R.N., Noll B.C., Guillot B., Samuel I., Aubert E. (2020). Aceclofenac and interactions analysis in the crystal and COX protein active site. J. Mol. Struct..

[31] Bitay E., Gergely A.L., Szabó Z.-I. (2023). Optimization and production of aceclofenac-loaded microfiber solid dispersion by centrifugal spinning. Pharmaceutics.

[32] Zheng Q., Unruh D.K., Hutchins K.M. (2022). Cocrystallization and thermal behaviors of the micropollutants gemfibrozil, aceclofenac, and bisphenol A. Cryst. Growth Des..

[33] Socrates G. (2004). Infrared and Raman Characteristic Group Frequencies.

[34] Thakur G., Singh A., Singh I. (2016). Chitosan-montmorillonite polymer composites: Formulation and evaluation of sustained release tablets of aceclofenac. Sci. Pharm..

[35] Suresh S., Gunasekaran S., Srinivasan S. (2014). Studies of the molecular geometry, vibrational spectra, Frontier molecular orbital, nonlinear optical and thermodynamics properties of Aceclofenac by quantum chemical calculations. Spectrochim. Acta Part A Mol. Biomol. Spectrosc..

[36] Dias C.J. (1996). Determination of a distribution of relaxation frequencies based on experimental relaxational data. Phys. Rev. B.

[37] Wübbenhorst M., van Turnhout J. (2002). Analysis of complex dielectric spectra. I. One-dimensional derivative techniques and three-dimensional modelling. J. Non-Cryst. Solids.

[38] Vogel H. (1921). Das Temperaturabhaengigkeitsgesetz der Viskositaet von Fluessigkeiten. Phys. Zeit..

[39] Fulcher G.S. (1925). Analysis of Recent Measurements of the Viscosity of Glasses. J. Am. Ceram. Soc..

[40] Tammann G., Hesse G.Z. (1926). Die Abhängigkeit der Viscosität von der Temperatur bie unterkühlten Flüssigkeiten. Z Anorg. Allg. Chem..

[41] Summerfield S. (1985). Universal low-frequency behaviour in the a.c. hopping conductivity of disordered systems. Philos. Mag. B-Phys. Conden. Mat. Stat. Mech. Electr. Opt. Magn. Prop..

[42] Johari G.P., Andersson O. (2006). On the nonlinear variation of dc conductivity with dielectric relaxation time. J. Chem. Phys..

[43] Psurek T., Hensel-Bielowka S., Ziolo J. (2002). Decoupling of the dc conductivity and (α-) structural relaxation time in a fragile glass-forming liquid under high pressure. J. Chem. Phys..

[44] Rodrigues A., Viciosa M.T., Danède F., Affouard F., Correia N.T. (2014). Molecular Mobility of Amorphous S-Flurbiprofen: A Dielectric Relaxation Spectroscopy Approach. Mol. Pharm..

[45] Adrjanowicza K., Wojnarowska Z., Wlodarczyk P., Kaminskia K., Paluch M., Mazgalski J. (2009). Molecular mobility in liquid and glassy states of Telmisartan (TEL) studied by Broadband Dielectric Spectroscopy. Eur. J. Pharm. Sci..

[46] Ruiz G.N., Romanini M., Hauptmann A., Loerting T., Shalaev E., Tamarit J.L., Pardo L.C., Macovez R. (2017). Genuine antiplasticizing effect of water on a glass-former drug. Sci. Rep..

[47] Diogo H.P., Ramos J.J.M., Viciosa M.T. (2023). The slow molecular mobility in the amorphous solid and supercooled liquid phases of brucine, an anti-cancer drug. A study by differential scanning calorimetry (DSC), thermostimulated currents (TSC) and dielectric relaxation spectroscopy (DRS). J. Mol. Liq..

[48] Figari G., Gonçalves J.L.M., Diogo H.P., Dionísio M., Farinha J.P., Viciosa M.T. (2023). Understanding Fenofibrate Release from Bare and Modified Mesoporous Silica Nanoparticles. Pharmaceutics.

[49] Ramos J.J.M., Diogo H.P. (2017). The determination of the glass transition temperature by thermally stimulated depolarization currents. Comparison with the performance of other techniques. Phase Trans..

[50] Williams G., Landel R., Ferry J.D. (1955). The temperature dependence of relaxation mechanisms in amorphous polymers and other glass forming liquids. J. Am. Chem. Soc..

[51] Starkweather H.W. (1981). Simple and complex relaxations. Macromolecules.

[52] Laredo E., Newman D., Pezzoli R., Müller A.J., Bello A. (2016). A Complete TSDC Description of Molecular Mobilities in Polylactide/Starch Blends from Local to Normal Modes: Effect of Composition, Moisture, and Crystallinity. J. Polym. Sci. Part B Polym. Phys..

[53] Gong L., Zhang X., Shi Y., Zhang L. (2012). Polarization temperature effect on liquid–liquid transition and space charge detrapping behavior in atactic polystyrene by thermally stimulated depolarization current. Polym. Bull..

[54] Mora E., Diogo H.P., Ramos J.J.M. (2015). The features of the normal mode relaxation as studied by thermally stimulated currents. Eur. Polym. J..

[55] Dudognon E., Bernès A., Lacabanne C. (2003). Dielectric manifestation of the liquid–liquid transition in poly(n-alkyl methacrylates). J. Macromol. Sci. Part B—Phys..

[56] Diogo H.P., Piedade M.F.M., Ramos J.J.M. (2021). Structure, thermal properties and molecular mobility in cholesteryl hydrogen phthalate: Different approaches to the crystal, the glassy crystal and the mesophase. J. Mol. Struct..

[57] Arrese-Igor S., Alegría A., Colmenero J. (2015). Dielectric relaxation of 2-ethyl-1-hexanol around the glass transition by thermally stimulated depolarization currents. J. Chem. Phys..

[58] Böhmer R., Gainaru C., Richert R. (2014). Structure and dynamics of monohydroxy alcohols—Milestones towards their microscopic understanding, 100 years after Debye. Phys. Rep..

[59] Correia N.T., Ramos J.J.M. (2000). On the cooperativity of the β-relaxation: A discussion based on dielectric relaxation and thermally stimulated depolarisation currents data. Phys. Chem. Chem. Phys..

[60] Johari G.P., Goldstein M. (1970). Viscous liquids and the glass transition. II. Secondary relaxations in glasses of rigid molecules. J. Chem. Phys..

[61] Johari G.P., Goldstein M. (1971). Viscous Liquids and the Glass Transition. III. Secondary relaxations in aliphatic alcohols and other nonrigid Molecules. J. Chem. Phys..

[62] Ramos J.J.M., Diogo H.P., Pinto S.S. (2007). Effect of physical aging on the Johari-Goldstein and relaxations of D-sorbitol: A study by thermally stimulated depolarization currents. J. Chem. Phys..

[63] Starzonek S., K-Ödzierska-Sar A., Drozd-Rzoska A., Szafran M., Rzoska S.J. (2018). Unique dynamic crossover in supercooled x,3-dihydroxypropyl acrylate (x = 1, 2) isomers mixture, *Eur*. Polym. J. E.

[64] Viciosa M.T., Ramos J.J.M., Diogo H.P. (2020). Thermal behavior and molecular mobility studies in the supercooled liquid and glassy states of carvedilol and loratadine. Int. J. Pharm..

[65] Ramos J.J.M., Correia N.T., Diogo H.P., Álvarez C., Ezquerra T.A. (2005). Slow relaxations in salicylsalicylic acid studied by dielectric techniques. J. Non-Cryst. Solids.

[66] Kurita R., Tanaka H. (2005). On the abundance and general nature of the liquid–liquid phase transition in molecular systems. J. Phys. Condens. Matter.

[67] Cao C., Tang W., Perepezko J.H. (2022). Liquid–liquid transition kinetics in D-mannitol. J. Chem. Phys..

[68] Brás A.R., Noronha J.P., Antunes A.M.M., Cardoso M.M., Schönhals A., Affouard F., Dionísio M., Correia N.T. (2008). Molecular motions in amorphous ibuprofen as studied by broadband dielectric spectroscopy. J. Phys. Chem. B.

[69] Yardimci H., Leheny R.L. (2006). Aging of the Johari-Goldstein relaxation in the glass-forming liquids sorbitol and xylitol, *J*. Chem. Phys..

[70] Vij J.K., Power G. (2011). Physical ageing and the Johari–Goldstein relaxation in molecular glasses. J. Non-Cryst. Solids.

[71] Prevosto D., Capaccioli S., Lucchesi M., Rolla P.A., Ngai K.L. (2004). Dynamics of supercooled and glassy dipropyleneglycol dibenzoate as functions of temperature and aging: Interpretation within the coupling model framework. J. Chem. Phys..

[72] Power G., Vij J.K., Johari G.P. (2006). Kinetics of spontaneous change in the localized motions of D-sorbitol glass. J. Chem. Phys..

[73] Ngai K., Paluch M. (2004). Classification of secondary relaxation in glass-formers based on dynamic properties. J. Chem. Phys..

[74] Kamińska E., Minecka A., Tarnacka M., Hachuła B., Kamiński K., Paluch M. (2020). Influence of annealing in the close vicinity of Tg on the reorganization within dimers and its impact on the crystallization kinetics of gemfibrozil. Mol. Pharm..

[75] Havriliak S., Negami S. (1967). A complex plane representation of dielectric and mechanical relaxation processes in some polymers. Polymer.

[76] Kremer F., Schönhals A. (2003). Broadband Dielectric Spectroscopy.

[77] Chen R., Kirsh Y. (1981). Analysis of Thermally Stimulated Processes.

[78] Teyssedre G., Lacabanne C. (1995). Some considerations about the analysis of thermostimulated depolarization peaks. J. Phys. D Appl. Phys..

[79] Ramos J.J.M., Viciosa M.T., Diogo H.P. (2019). Thermal behaviour of two anti-inflammatory drugs (celecoxib and rofecoxib) and slow relaxation dynamics in their amorphous solid state. Comparison between the dynamic fragility obtained by dielectric spectroscopy and by thermostimulated currents. Mol. Phys..

