# Supplementary material for: Dynamic Behavior of the Glassy and Supercooled Liquid States of Aceclofenac Assessed by Dielectric and Calorimetric Techniques
Source: Molecules. 2025 Feb 4;30(3):681. doi: 10.3390/molecules30030681 (PMC11820318; doi:10.3390/molecules30030681)
Supplement: Supplementary file 1 [file molecules-30-00681-s001.zip › molecules-3416993-supplementary.pdf]

Supplementary Information

Dynamic behavior of the glassy and supercooled liquid states of Aceclofenac assessed by dielectric and calorimetric techniques

M. Teresa Viciosa[1,*], Joaquim J. Moura Ramos[1], Ana Rosa García[1,2] and Hermínio P. Diogo[1,*]

[1] Centro de Química Estrutural, Institute of Molecular Sciences, Instituto Superior Técnico, Universidade de Lisboa, Av. Rovisco Pais 1049-001 Lisboa, Portugal; mouraramos@tecnico.ulisboa.pt (J.J.M.R.)

[2] Departamento de Química e Farmácia, FCT, Universidade do Algarve, Campus de Gambelas, Faro 8000-139, Portugal; argarcia@ualg.pt (A.R.G.)

* Correspondence: teresaviciosa@tecnico.ulisboa.pt, hdiogo@tecnico.ulisboa.pt

Table of contents

1. DSC..2
2. FTIR..6
3. DRS..7
4. TSDC..8

1. DSC

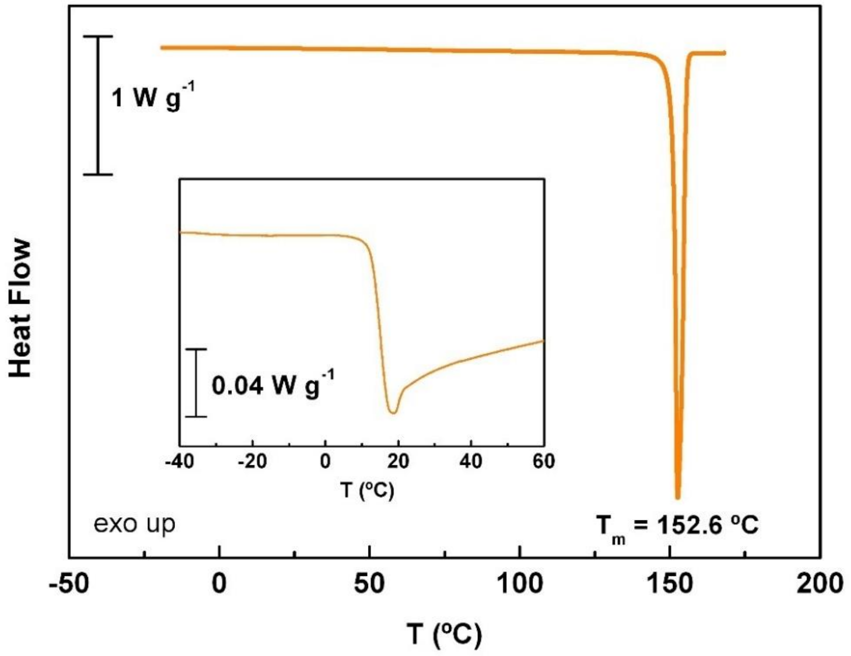

Figure S1. Thermograms obtained by heating on a crystalline fresh ACF sample at 5 °C·min^{-1} (main figure) and subsequent cooling to -80 °C at 10 °C·min^{-1} (inset).

In Figure S2, the glass transition step is observed, shifting to lower temperatures ($T_{g\text{-}on}$ = 2.7 °C) compared to the fresh amorphized sample ($T_{g\text{-}on}$ = 9.2 °C). Additionally, the sample undergoes cold crystallization with a maximum of 94.6 °C. The corresponding melting temperature was detected at T = 115.6 °C, which is notably lower than that of crystalline polymorph I (fresh sample). This result strongly suggests the formation of a new polymorphic form.

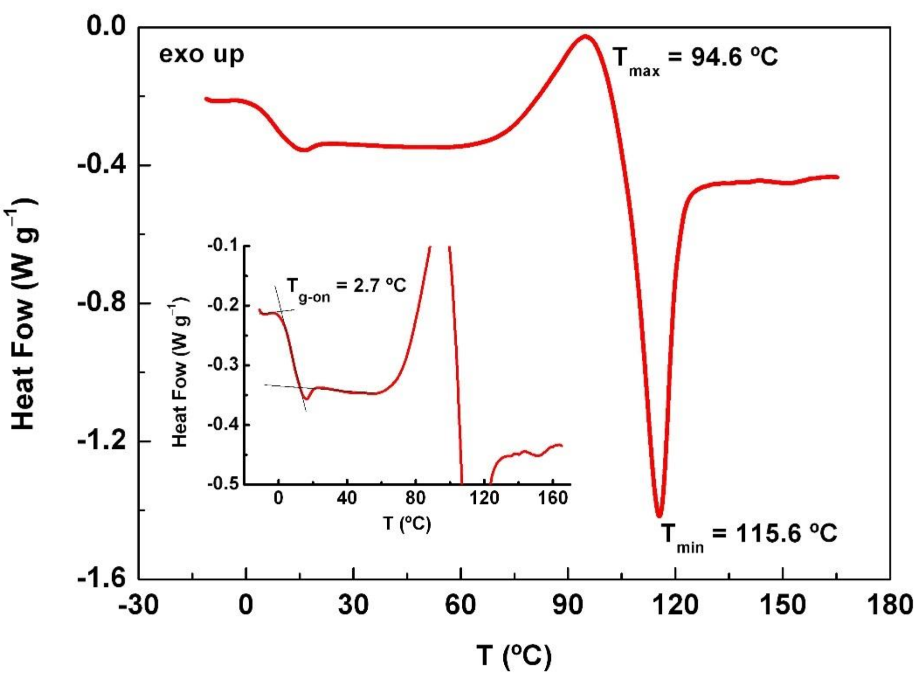

Figure S2. Thermogram obtained by heating an amorphous ACF sample at 15 °C·min^{-1}. From low to high temperatures, it shows the signature of the glass transition (~3 °C), an exothermic peak representing crystallization (~95 °C), followed by an endothermic peak corresponding to the melting of the crystal (~116 °C). The inset shows a magnification of the glass transition signal.

Hot-stage microscopy was used to directly observe the crystals formed by cold crystallization (heating ramp of 5 °C·min^{-1}) after the melting of phase I and cooling below T_g. Some images taken at 90 °C are shown in Figure S3.

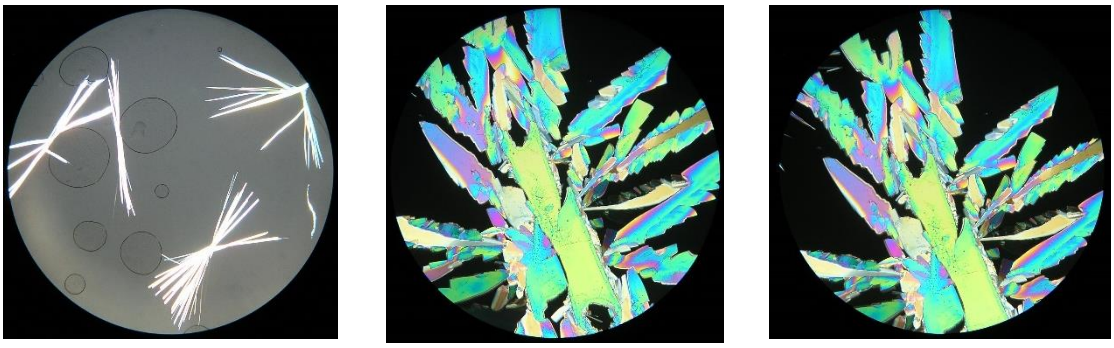

100X 250X 250X

Figure S3. Microstructure of ACF formed during heating from -30 °C at 5 °C·min^{-1} (images taken at 90 °C). Equipment: Olympus BX51 optical microscope equipped with a Linkam LTS360 liquid nitrogen-cooled cryostat.

Further research is needed to gain a better understanding of this crystalline form, which has yet to be fully studied, and to definitively determine the optimal experimental conditions for its synthesis—something we aim to explore in the near future.

Table S1. Real heating rates and the temperature onset of the glass transition signal are depicted in Figure 2 of the manuscript.

***q*+ (°C·min^{-1})**	**T_{g-on} (°C)**
1.9	9.85
2.3	10.67
2.8	10.90
3.3	11.39
3.8	11.46
4.2	11.95
4.7	12.06
5.7	12.34
6.6	12.40
7.6	12.66
8.5	12.83
9.5	13.16
10.4	13.44
11.4	13.54
12.3	13.59
13.2	13.85
14.2	14.11
14.2	14.14

2. FTIR

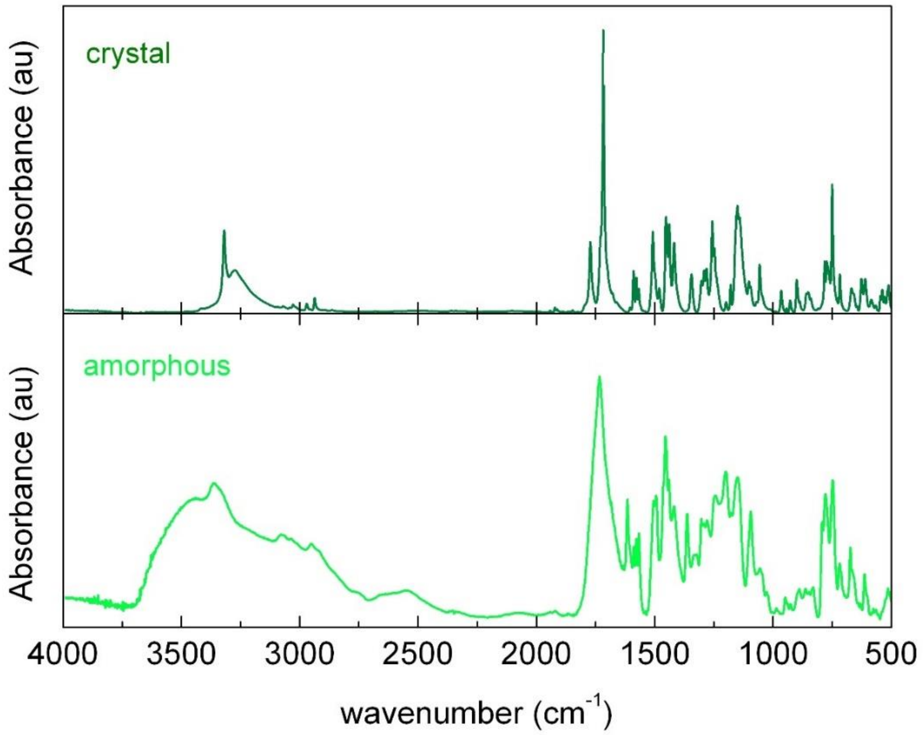

Figure S4. FTIR spectra of crystalline and amorphous ACF samples.

3. DRS

The reduction of the dc conductivity from the ε''(f) spectrum by the derivative approximation, $\varepsilon'_{der} = -\frac{\pi}{2}\frac{d\varepsilon'}{dln\omega}$, allows a better resolution of the Debye peak located at lower frequencies than those of the α one (green peak in Figure S6).

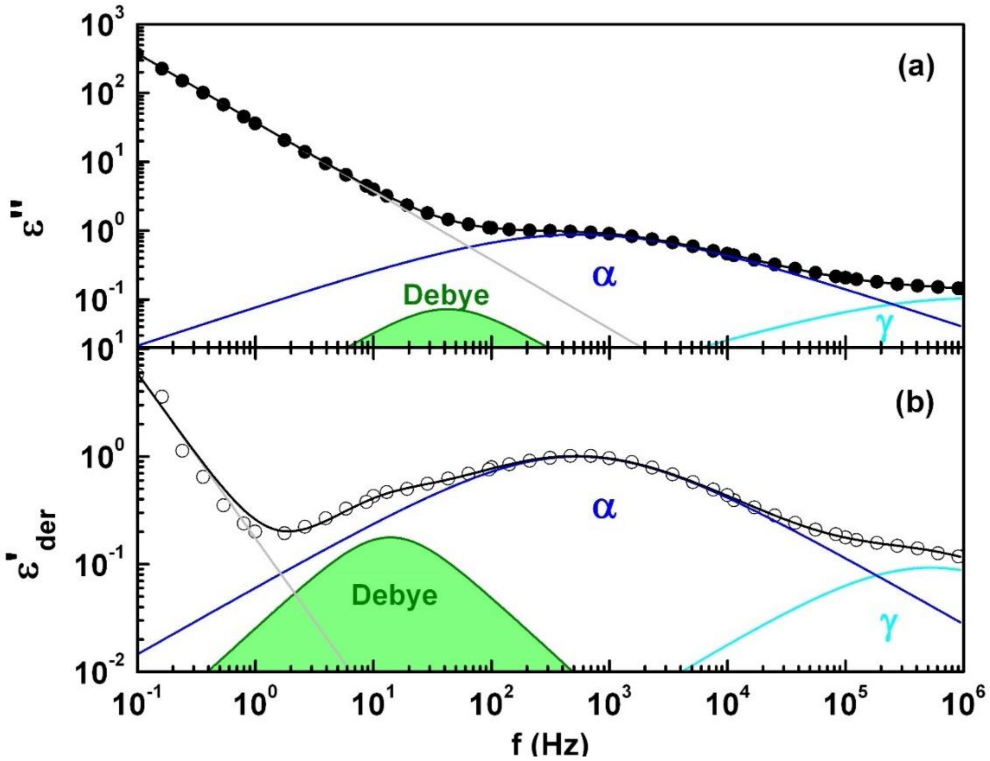

Figure S5. Isothermal spectrum of amorphous ACF collected at 28 °C: (**a**) ε'', and (**b**) ε'_{der}. The colored lines represent the individual HN fitting functions, while the black line shows the overall fitted curve.

4. TSDC

Using the same experimental procedure to obtain the amorphous state (melting followed by cooling), different results were observed regarding the intensity of the LL peak, while the intensity of the main relaxation peak remained nearly unaffected. For example, the degree of cooling from the liquid (i.e., the final temperature reached at the end of the cooling step) influences the intensity, as shown in the following figure, while the temperature of the α peak remains virtually unchanged.

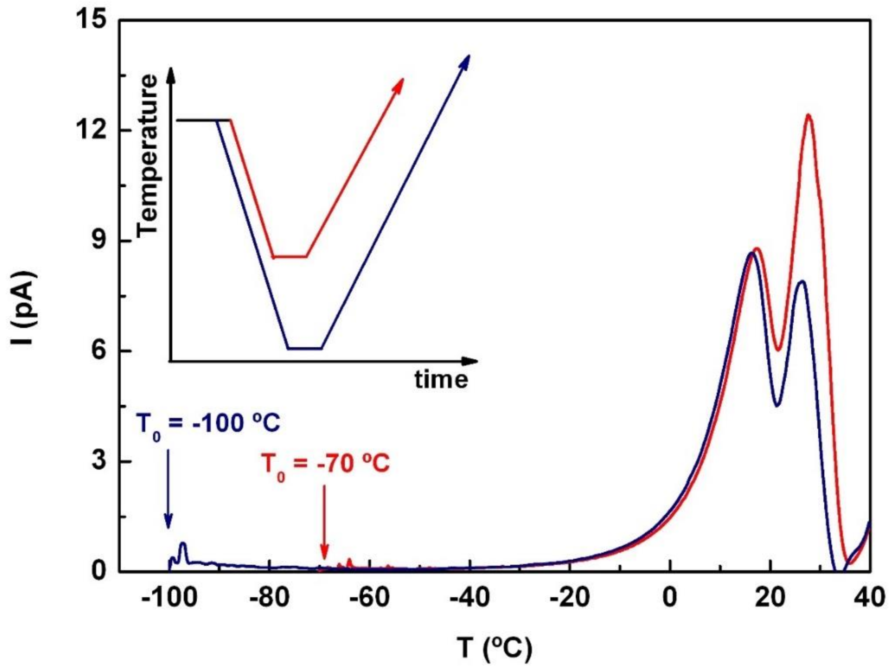

Figure S6. TSDC global results showing the α-relaxation and the relaxation above T_g. In both situations the polarization temperatures were T_P = 33 °C; the electric field applied, and the heating rate were respectively 250 V·mm^{-1} and 8 °C·min^{-1}. T_0 = -70 °C (red) and T_0 = -100 °C (black). Inset: schematic representation of global TSDC experiment.

The final (or structural) state of an amorphization system depends on the experimental conditions (thermal history) used to achieve it and is not fixed. Since it is a non-equilibrium state, the kinetic nature of the transition toward equilibrium plays a crucial role. Fortunately, in many systems, the TSC technique has proven sensitive enough to detect even small structural changes, at least qualitatively, across different amorphous stages, acting as a probe that generates distinct signatures.
